# Reduction in Antimicrobial Use and Resistance to *Salmonella*, *Campylobacter*, and *Escherichia coli* in Broiler Chickens, Canada, 2013–2019

**DOI:** 10.3201/eid2709.204395

**Published:** 2021-09

**Authors:** Laura Huber, Agnes Agunos, Sheryl P. Gow, Carolee A. Carson, Thomas P. Van Boeckel

**Affiliations:** Auburn University, Auburn, Alabama, USA (L. Huber); ETH Zürich, Zürich, Switzerland (L. Huber, T.P. Van Boeckel);; Public Health Agency of Canada, Guelph, Ontario, Canada (A. Agunos, C.A. Carson);; Public Health Agency of Canada, Saskatoon, Saskatchewan, Canada (S.P. Gow);; Center for Disease Dynamics, Economics & Policy, Washington, DC, USA (T.P. Van Boeckel)

**Keywords:** foodborne pathogens, antimicrobial resistance, antimicrobial use, food safety, animal production, *Salmonella*, *Campylobacter*, *Escherichia coli*, broiler chickens, bacteria

## Abstract

Antimicrobial use contributes to the global rise of antimicrobial resistance (AMR). In 2014, the poultry industry in Canada initiated its Antimicrobial Use Reduction Strategy to mitigate AMR in the poultry sector. We monitored trends in antimicrobial use and AMR of foodborne bacteria (*Salmonella*, *Escherichia coli*, and *Campylobacter*) in broiler chickens during 2013 and 2019. We quantified the effect of antimicrobial use and management factors on AMR by using LASSO regression and generalized mixed-effect models. AMR in broiler chickens declined by 6%–38% after the decrease in prophylactic antimicrobial use. However, the withdrawal of individual compounds, such as cephalosporins and fluoroquinolones, prompted an increase in use of and resistance levels for other drug classes, such as aminoglycosides. Canada’s experience with antimicrobial use reduction illustrates the potential for progressive transitions from conventional antimicrobial-dependent broiler production to more sustainable production with respect to antimicrobial use.

In Canada, foodborne pathogens cause an estimated 4 million cases of human illness, 11,600 hospitalizations, and 238 deaths each year (*1*). *Escherichia coli*, *Campylobacter*, and *Salmonella* are the foodborne zoonotic pathogens most frequently associated with infections from poultry products (*2*). Antimicrobial drugs have been used in ovo, feed, or water to prevent or treat commonly occurring diseases of poultry and to enable gains in productivity on farms (*3*,*4*). However, use of antimicrobial drugs contributes to the development of antimicrobial resistance (AMR). In humans, treatment of salmonellosis with antimicrobial drugs is often unnecessary but may be life-saving in the case of invasive infections (*5*). The rise of AMR progressively reduces the number of antimicrobial drug options available to treat infections, which has important consequences for human health but also for the long-term viability of the production of animals (*6*–*8*).

In 2005, the Canadian Integrated Program for Antimicrobial Resistance Surveillance (CIPARS) reported an increasing frequency of resistance to ceftiofur, a veterinary third-generation cephalosporin (*9*), in *Salmonella enterica* serovar Heidelberg isolates from retail chicken and humans (*10*). In response, broiler chicken producers in Québec Province voluntarily eliminated the extra-label use of ceftiofur through injection (in ovo or subcutaneously) in hatcheries (*11*). By 2006, this measure led to a reduction in prevalence of ceftiofur-resistant *Salmonella* Heidelberg in retail chicken and humans (*8*). In a concerted effort to mitigate AMR and to reduce overall antimicrobial use (AMU), a stewardship program called the Antimicrobial Use Reduction Strategy was initiated in 2014 by the poultry industry. The first objective of this program was the elimination of the preventive use of Health Canada’s Veterinary Drugs Directorate’ category I antimicrobials (*12*), including third-generation cephalosporins (e.g., ceftiofur) and fluoroquinolones, which was accomplished in 2014 (*13*). Subsequently, the goal was to eliminate the preventive use of category II antimicrobials (e.g., aminoglycosides, lincosamides-aminocyclitols, macrolides, penicillin, and trimethoprim/sulfonamide combinations), which was accomplished in the end of 2018. The third phase was to include the elimination of the preventive use of category III antimicrobials (e.g., bacitracins and tetracyclines) by the end of 2020 (*13*). This third step has been postponed pending further consultation with producers, an assessment of overall bird health and welfare from implementation of the first 2 phases, and a more fulsome evaluation of the production outcomes.

In our study, we used farm-level AMU and AMR time series data from CIPARS (2013–2019) to identify how changes in AMU have affected AMR in *E. coli*, *Campylobacter*, and *Salmonella* isolates from broiler chicken farms in Canada. The specific goals were to assess trends in AMR by province during 2013–2019, identify farm-management factors affecting AMU and AMR, and examine the association between route of antimicrobial administration (injections, water, or feed) and the frequency of multidrug resistance (defined as resistance to >2 antimicrobial classes).

## Material and Methods

### Study Design and Data Collection

We collected AMU and AMR information at the farm level through a network of poultry veterinarians (n = 17) who were assigned to producers (n = 97–147, depending on the year) in the 5 major poultry-producing provinces of Canada: British Columbia, Alberta, Saskatchewan, Ontario, and Québec (*14*). Participating producers signed an informed consent form, which was administered by the veterinarian. We obtained information on farm-level AMU and farm demographics by using a questionnaire and collected fecal samples for bacterial recovery and antimicrobial-susceptibility testing. We collected samples according to the formula for detection of AMR in a population of >1,000 individuals (n = ln α / ln [1 – minimum expected prevalence]; α = 0.05) (*15*), according to the routine CIPARS/FoodNet Canada farm sampling strategy. We divided each barn from each farm in 4 quadrants, and we collected 10–15 fresh fecal droppings from each quadrant. We pooled the samples from each quadrant and selected randomly 1 isolate per pooled sample for all *E. coli*, *Salmonella*, and *Campylobacter* for further analysis. Each year, we sampled 1 flock of preharvest broilers (>30 days old) that had been randomly selected from each production unit. We administered questionnaires to record flock characteristics, including hatchery or province and country of origin of the hatching eggs or chicks, breed, production system (conventional or antimicrobial-free), age, and estimated weight of birds at preharvest sampling. We collected detailed AMU information, including the quantity of antimicrobial active ingredients administered, routes of administration (in ovo or subcutaneous injections at the hatchery, feed, and water) and primary reasons for use of antimicrobial (prophylaxis, growth promotion, or disease treatment). We also collected information on biosecurity, health status, and vaccination history (questionnaires were published elsewhere [*16*] as supplemental material).

### Bacteria Isolation and Susceptibility Testing

When an isolate of each bacterial species of interest (*Salmonella*, *E. coli* and *Campylobacter*) was identified, we saved that isolate and tested it for susceptibility. We conducted antimicrobial-susceptibility testing by using routine CIPARS methodology (*14*). We performed automated broth microdilution by using Sensititre (ThermoFisher Scientific, https://www.thermofisher.com) using the CMV4AGNF panel for *Salmonella* and *E. coli* and the CAMPY plates for *Campylobacter.* Plate configurations were designed by the US National Antimicrobial Resistance Monitoring System. We applied Clinical and Laboratory Standards Institute breakpoint guidelines (*17*,*18*) (Appendix Table 1). According to routine CIPARS/National Antimicrobial Resistance Monitoring System methods, we classified isolates with intermediate susceptibility patterns as susceptible. According to CIPARS AMR testing methods, we sued no selective media in this study.

### Statistical Analysis

The number of antimicrobial classes each isolate was resistant to (nC) was the main outcome in the regression models. We evaluated the effect of covariates on the nC by using a 2-step procedure. First, we used a LASSO regression to select a subset of risk factors to be included in the generalized models (Appendix Table 2). Second, we ran a mixed-effect model with veterinarian and flock identification as random effects in all models. We cross-validated the models by dividing the dataset into 3 validation sets.

The term “ideal method for cleaning and disinfection” refers to the method recommended by the World Organisation for Animal Health (OIE) (*19*) aimed at reducing infectious pathogens in animal premises. This method consists of dry cleaning (i.e., removing of all equipment and brushing and scraping all surfaces), followed by a warm water (60°C) wash and application of a disinfectant to reduce microbial populations and carry over of pathogens to the next production cycle. For production system categories, the term “antimicrobial-free”(in contrast with “conventional” refers to farms that were not exposed to nationally defined medically important antimicrobials (*20*) or farms that have a reduced AMU program (i.e., one that may allow use of chemical coccidiostats, according to guidelines [*21*], or ionophores). We estimated AMU at the flock level in milligrams of antimicrobial active ingredient per kilogram broiler chicken biomass (mg/kg) by summing of all antimicrobials reportedly used in the flock from all routes of administration and dividing by the live animal biomass (e.g., birds at risk multiplied by the average preslaughter live weight) (*22*).

We compared the model fit between models by using the Akaike information criteria and the likelihood ratio test. We performed post hoc pairwise testing of mean flock differences in nC among groups of disinfection method, use of antimicrobials at the hatcheries, year, and province by using Tukey’s multiple comparison test.

We quantified the trends of antimicrobial use (Appendix Figures 2–4 for *Salmonella*, 8–10 for *E. coli*, and 14–16 for *Campylobacter*), and the association between resistance for individual antimicrobial classes (Appendix Figures 5–7 for *Salmonella*, 11–13 for *E. coli*, and 17–19 for *Campylobacter*) by using mixed-effect logistic regression models for each bacterial species. We conducted all statistical analysis in RStudio 1.2.5033 (https://www.rstudio.com) and defined statistical significance as p<0.05.

## Results

### Temporal Differences, Regional Differences, and Factors Associated with AMR

For *Salmonella*, the nC an isolate was resistant to in 2018 was 0.9 times lower than the nC an isolate was resistant to in 2013 (p<0.001); however, the nC an isolate was resistant to in 2019 was 1.6 times higher than in 2013 (p = 0.045), given that other variables were held constant in the model. In individual provinces, compared with the value for Alberta, the nC an isolate was resistant to was 1.7 times higher in British Columbia (p = 0.007), 1.8 times higher in Ontario (p = 0.002), 3.8 times higher in Québec (p<0.001), and 1.9 times higher in Saskatchewan (p = 0.009). For every 1-unit increase in antimicrobial injected in ovo (mg/kg) in the hatcheries, the national nC an isolate was resistant to increased by 3.4 (p = 0.02). Posthoc (Tukey test) showed that Ontario (p = 0.015) and Québec (p<0.001) had a significantly higher mean nC that an isolate was resistant to compared with Alberta; Québec also had a significantly higher mean nC that an isolate was resistant to than British Columbia and Ontario across all years (p<0.001 for both provinces) (Table 1). The antibiotic-free flocks (n = 286) were not different from conventional flocks (n = 1,612) in the nC an isolate was resistant to (Table 1). However, prevalence of *Salmonella* Heidelberg was statistically significantly higher at conventional farms (Appendix Figure 1). Using the ideal method of disinfection, which that entails dry and wet cleaning followed by the application of a disinfectant, was not a significant factor in the nC a *Salmonella* isolate was resistant to. However, significantly higher prevalence of *Salmonella* Heidelberg and Kentucky (Appendix Figure 1) was found in flocks that did not use the ideal method of disinfection.

**Table 1:** Incidence rate ratio of *Salmonella* nC from LASSO-penalized generalized mixed-effects Poisson model in a study of antimicrobial use and in broiler chickens, Canada, 2013–2019*

Variable	Incidence rate ratio	2.5% CI	97.5% CI	p value
Intercept	0.224851	0.1326975	−0.3810016	2.92 × 10^-8^†
Production system (referent comparison factor: conventional)				
Antimicrobial-free‡	1.456588	0.9917592	2.1392781	0.05514
Disinfection system (referent comparison factor: no use of the ideal method of disinfection)				
Use of ideal disinfection	0.8947851	0.6969602	1.1487606	0.38316
Continuous variables of antimicrobial use (mg/kg)				
Injections (in ovo or subcutaneous§)	3.3926736	1.1860941	9.704318	0.02271†
Through feed	1.0030552	1.0004128	1.0057047	0.02341†
Through water	1.0005486	0.9947253	1.006406	0.85389
Sample collection year (referent comparison year: 2013)				
2014	0.9904373	0.6355585	1.5434709	0.96614
2015	1.0475486	0.6851365	1.6016635	0.83021
2016	1.0912259	0.7028907	1.6941097	0.69726
2017	0.9097193	0.5821923	1.4215049	0.67777
2018	0.9869455	0.6351112	1.5336864	9.53 × 10^-1^†
2019	1.548854	1.0091025	2.3773092	0.04534†
Province (referent comparison province: Alberta)				
British Columbia	1.6846635	1.1510546	2.465644	0.00728†
Ontario	1.8199429	1.2502213	2.6492848	0.00177†
Québec	3.7534112	2.4943597	5.6479808	2.24 × 10^-1^†
Saskatchewan	1.9772529	1.1775379	3.3200878	0.00994†

*nC, number of antimicrobial classes to which each isolate was resistant. †Statistically significant (p<0.05). ‡Antimicrobial-free flocks were not exposed to medically important antimicrobials through any route of administration. §Subcutaneous route in young chicks at the hatchery.

For *E. coli*, nationally, during 2018 and 2019, the nC an isolate was resistant to was 0.9 (in 2018, p = 0.015) and 0.8 (in 2019, p<0.001) times lower than the nC an isolate was resistant to in 2013 after controlling for other variables (Table 2). The nC an isolate was resistant to was 1.2 times higher in British Columbia (p = 0.032) and 1.4 times higher in Québec (p<0.001) than the nC an isolate was resistant to in Alberta; in Saskatchewan, the nC an isolate was resistant to was 0.5 times lower than in Alberta (p<0.001). Posthoc (Tukey test) examination demonstrated that the provinces of British Columbia, Ontario, Québec, and Saskatchewan had a significantly higher mean nC an isolate was resistant to compared with Alberta; Québec also had a significantly higher mean nC an isolate was resistant to than the means for British Columbia and Ontario. In 2019, we observed a significantly lower nC an isolate was resistant to than in 2013 (p = 0.002), 2014 (p = 0.002), 2015 (p = 0.012), and 2016 (p = 0.014) (Table 2). The antibiotic-free status of the flock and ideal method of disinfection were not significant factors in the nC to which an *E. coli* isolate was resistant.

**Table 2:** Incidence rate ratio of *Escherichia coli* nC from LASSO-penalized generalized mixed-effects Poisson model in a study of antimicrobial use and in broiler chickens, Canada, 2013–2019*

Variable	Incidence rate ratio	2.50% CI	97.50% CI	p value
Intercept	1.5740809	1.3050913	1.8985113	2.09 × 10^-6^†
Production system (referent comparison factor: conventional)				
Antimicrobial-free‡	1.0275338	0.9170807	1.1512897	0.63969
Ideal disinfection method (referent comparison factor: no use of ideal method)				
Use of ideal disinfection	1.0133377	0.9418401	1.0902627	0.722652
Continuous variables of antimicrobial use (mg/kg)				
Injections (in ovo or subcutaneous§)	1.3588476	0.9911794	1.8628985	0.056785
Through feed	1.0015582	1.0008262	1.0022907	2.99 × 10^-5^†
Through water	1.0032516	1.0019576	1.0045473	8.23 × 10^-7^†
Sample collection year (referent comparison year: 2013)				
2014	0.8881768	0.7850343	1.0048707	0.05972
2015	0.9555537	0.8431346	1.0829621	0.476499
2016	0.9458207	0.8349598	1.071401	0.381178
2017	0.9144284	0.8066086	1.0366604	0.162256
2018	0.8545609	0.7523902	0.9706058	0.015553†
2019	0.7705043	0.6770116	0.8769079	7.81 × 10^-5^†
Province (referent comparison province: Alberta)				
British Columbia	1.2229891	1.0173109	1.4702509	0.032138†
Ontario	0.9922909	0.8315428	1.1841136	0.931604
Québec	1.3924895	1.1564315	1.6767333	0.000477†
Saskatchewan	0.4997466	0.3844197	0.649672	2.20 × 10^-7^†

*nC, number of antimicrobial classes to which each isolate was resistant. †Statistically significant (p<0.05). ‡Antimicrobial-free flocks were not exposed to medically important antimicrobials through any route of administration. §Subcutaneous route in young chicks at the hatchery.

For *Campylobacter*, in 2016, the nC to which an isolate was resistant was 0.4 times lower than the nC for 2013, given that other variables were held constant in the model (p = 0.03). Posthoc (Tukey test) comparison shows that 2016 (p = 0.008) and 2018 (p = 0.037) had a significantly lower mean nC to which an isolate was resistant than the value for 2015 (Table 3). The antibiotic-free status of the flock and ideal method of disinfection were not significant factors in the nC to which a *Campylobacter* isolate was resistant.

**Table 3:** Incidence rate ratio of *Campylobacter* nC from LASSO-penalized generalized mixed-effects Poisson model in a study of antimicrobial use and in broiler chickens, Canada, 2013–2019*

Variable	Incidence rate ratio	2.50% CI	97.50% CI	p value
Intercept	0.277081	0.1054967	0.7277371	0.00919†
Production system (referent comparison factor: conventional)				
Antimicrobial-free‡	0.60892	0.2994255	1.2383169	0.17076
Ideal disinfection method (referent comparison factor: no use of ideal method)				
Use of ideal disinfection	1.3043882	0.7766548	2.190714	0.31513
Continuous variable of antimicrobial use (mg/kg)				
Injections (in ovo or subcutaneous§)	1.7448076	0.1650191	18.4484971	0.64363
Through feed	0.9979396	0.9923108	1.0036003	0.4748
Through water	0.996652	0.9806929	1.0128707	0.68386
Sample collection year (referent comparison year: 2013)				
2014	0.7218903	0.3138241	1.6605658	0.44323
2015	1.7590844	0.8024374	3.8562237	0.15843
2016	0.3714697	0.1493034	0.9242233	0.03322†
2017	0.8334422	0.3732234	1.8611531	0.65669
2018	0.5029853	0.2104181	1.2023406	0.12221
2019	0.6468213	0.2868202	1.4586765	0.29368
Province (referent comparison province: Alberta)				
British Columbia	1.6638783	0.9144055	3.0276404	0.09551
Ontario	1.5284206	0.7983312	2.9261911	0.20045
Québec	2.0744067	0.9336274	4.6090798	0.07323
Saskatchewan	1.8708334	0.5308387	6.5933731	0.32975

*nC, number of antimicrobial classes to which each isolate was resistant. †Statistically significant (p<0.05). ‡Antimicrobial-free flocks were not exposed to medically important antimicrobials through any route of administration. §Subcutaneous route in young chicks at the hatchery.

### Prevalence of Resistance by Antimicrobial Drug

Prevalence of resistance remained <15% (Appendix Table 1) for 10 of 13 tested antimicrobials for *Salmonella* isolates (n = 1,898), 7 of 13 tested antimicrobials for *E. coli* isolates (n = 3,671), and 5 of 8 tested antimicrobials for *Campylobacter* isolates (n = 769). The prevalence of *Salmonella* isolates resistant to tetracycline was 44.7% (95% CI 42.5%–46.9%) and to streptomycin was 43.6% (95% CI 41.3%–45.8%) (Appendix Table 1). Moreover, prevalence of *E.coli* isolates resistant to tetracycline was 46.8% (95% CI 45.2%–48.4%), to streptomycin was 46.3% (95% CI 44.7%–47.9%), to sulfisoxazole was 39.4% (95% CI 37.8%–41.0%), to ampicillin was 40.5% (95% CI 38.9%–42.1%), to gentamicin was 18.4% (95% CI 17.2%–19.7%), and to trimethoprim/sulfamethoxazole was 16.1% (95% CI 14.9%–17.3%) (Appendix Table 1). The prevalence of *Campylobacter* isolates resistant to tetracycline was 38.8% (95% CI 35.3%–42.2%), to ciprofloxacin was 16.5% (95% CI 13.9%–19.1%), and to nalidixic acid was 16.4% (95% CI 13.8%–19.0%) (Appendix Table 1).

### Temporal Trend of AMR by Antimicrobial Class

**Figure 1:**
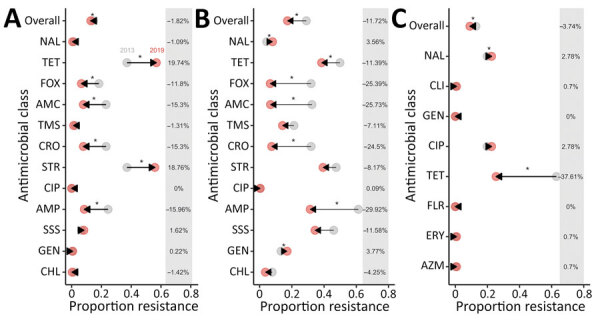
Change in mean proportion of antimicrobial resistance *in Salmonella* (A), *Escherichia coli* (B), and *Campylobacter* (C) in in broiler chickens, overall and by drug class, Canada, 2013–2019. Arrows represent directionality of proportion change in resistance from 2013 (gray) to 2019 (red) for each of the antimicrobial classes. Differences in proportion resistance from 2013 to 2019 are presented on the right side of each graph. Asterisks indicate p<0.05 as determined by mixed-effects logistic regression, including year and antimicrobial use (in ovo or through subcutaneous injection, water, and feed) as fixed effects and flock and veterinarian identification as random effects. AMC, amoxicillin/clavulanic acid; AMP, ampicillin; AZM, azithromycin; CHL, chloramphenicol; CIP, ciprofloxacin; CLI, clindamycin; CRO, ceftriaxone; ERY, erythromycin; FLR, florfenicol; FOX, cefoxitin; GEN, gentamycin; NAL, nalidixic acid; SSS, sulfisoxazole; STR, streptomycin; TET, tetracycline; TMS, trimethropim/sulfonamides.

**Figure 2:**
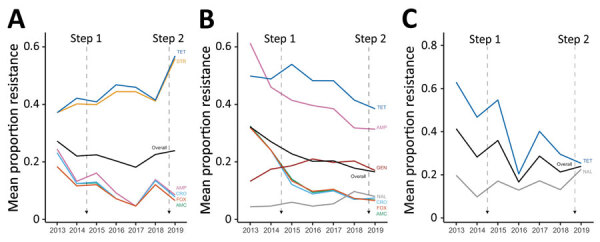
Significant changes (p<0.05) in mean proportion of antimicrobial resistance *in Salmonella* (A), *Escherichia coli* (B), and *Campylobacter* (C) in in broiler chickens, by antimicrobial class, Canada, 2013–2019. Step 1 is the elimination of the preventive use of category I antimicrobials in May 2014 (third-generation cephalosporins and fluoroquinolones) as part of Antimicrobial Use Reduction Strategy stewardship program. Step 2 is the elimination of the preventive use of category II antimicrobials in the end of 2018 (aminoglycosides, lincosamides, macrolides, penicillin, quinolones, streptomycin, and trimethoprim/sulfonamide combinations). Step 3, which was the elimination of the preventive use of category III antimicrobials (e.g., bacitracins and tetracyclines) by the end of 2020, is not represented in the figure. AMC, amoxicillin/clavulanic acid; AMP, ampicillin; CRO, ceftriaxone; FOX, cefoxitin; GEN, gentamycin; NAL, nalidixic acid; STR, streptomycin; TET, tetracycline.

For *Salmonella*, we observed a significant decrease in the mean resistance rates across all antimicrobial drugs included in the panel (1.8%), as well as individually to to cefoxitin (11.8%), amoxicillin/clavulanic acid (15.3%), ceftriaxone (15.3%), and ampicillin (15.9%) during 2013–2019. However, AMR rose significantly in streptomycin (18.8%) and tetracycline (19.7%) during the same period (Figures 1, 2). For *E. coli*, we observed a significant decrease in resistance overall (11.7%), as well as individually to tetracycline (11.4%), cefoxitin (25.4%), amoxicillin/clavulanic acid (25.7%), ceftriaxone (24.5%), and ampicillin (29.9%), whereas resistance to gentamicin (3.8%) and nalidixic acid (3.6%) increased (Figures 1, 2). For *Campylobacter*, we observed a significant decrease in overall (3.7%) resistance and to tetracycline (37.6%), but we observed a significant increase in nalidixic acid resistance (2.8%) (Figures 1, 2).

### Temporal Trend of Antimicrobial Use by Class

**Figure 3:**
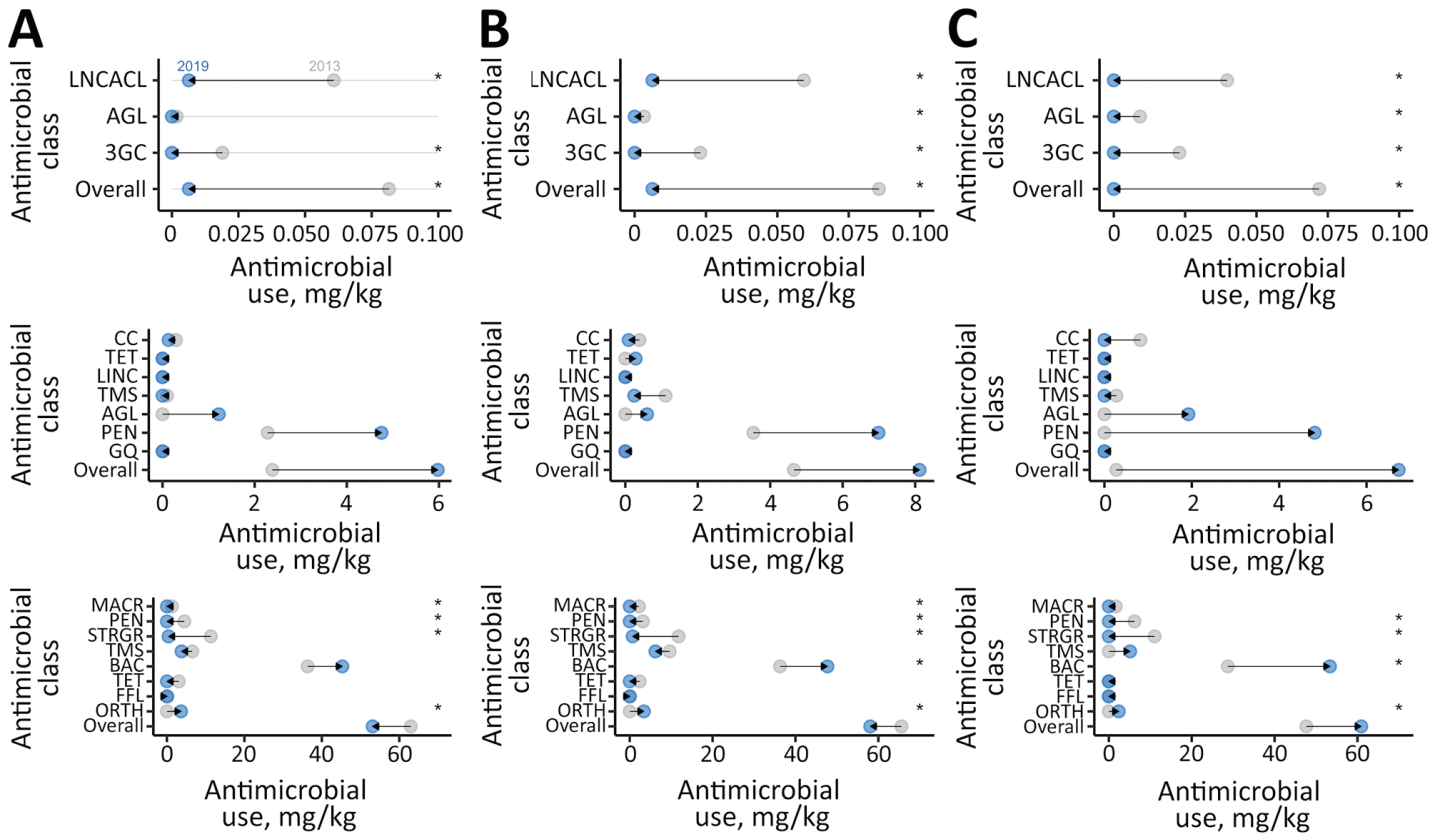
Mean antimicrobial use among broiler chicken flocks by bacterial species and route of administration, Canada, 2013–2019. A) *Salmonella*; B) *Escherichia coli*; C) *Campylobacter.* Route of administration in each panel: top, in ovo or subcutaneous injections; middle, water; bottom, feed. Arrows represent directionality of the antimicrobial use change from 2013 (gray) to 2019 (blue) of each antimicrobial class. Asterisks indicate p<0.05 as determined by a generalized mixed-effects model, including year as fixed effects and flock and veterinarian identification as random effects. AGL, aminoglycoside; BAC, bacitracin; CC, chemical coccidiostats; FFL, flavophospholipid; FQ, fluoroquinolone; LINC, lincomycin; LNCACL, lincosamides; MACR, macrolide; ORTH, orthomycin; PEN, penicillin; STRGR, streptogramin; TET, tetracycline; TMS, trimethropim/sulfonamides; 3GC, third-generation cephalosporin.

**Figure 4:**
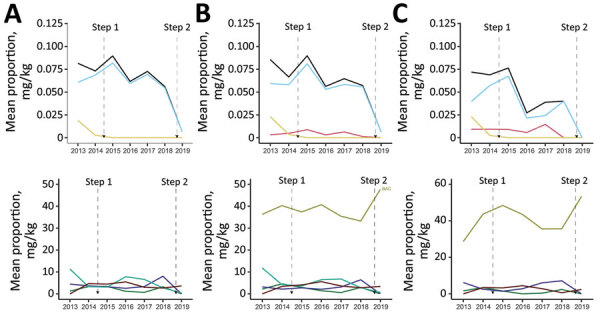
Mean antimicrobial use administered in ovo or subcutaneously at broiler chicken hatcheries or through feed, by isolation of bacterial species, Canada, 2013–2019. A) *Salmonella*; B) *Escherichia coli*; C) *Campylobacter.* Route of administration in each panel: top, in ovo or subcutaneous injections; bottom, feed. Mean antimicrobial use is color coded: lincosamides, in light blue; overall, in black; third-generation cephalosporins, in yellow; orthosomycins, in brown; penicillins, in purple; streptomycin, in cyan; and macrolides, in green. Antimicrobials are represented only if significantly (p<0.05) changing over time. The antimicrobial use trend through water is not represented because no statistically significant differences were found.

In flocks where *Salmonella* was isolated, we observed a significant decrease in overall AMU, use of lincosamide-aminocyclitol combinations, and use of third-generation cephalosporins through injection (in ovo or subcutaneous routes) during 2013–2019 (Figures 3, 4; Appendix Figures 3, 4). For feed, we observed a statistically significant decrease in the use of macrolides, penicillins, streptogramins, but we observed a significant increase in the use of orthosomycins (Figures 3, 4; Appendix Figure 4). In flocks where *E. coli* was isolated, we observed a significant decrease in injectable antimicrobials during 2013–2019 (Figures 3, 4; Appendix Figure 8). We observed a decrease in the use of penicillins and streptogramins and an increase in the use of bacitracins and orthosomycins through feed over time (Figures 3, 4; Appendix Figure 10). In flocks where *Campylobacter* was isolated, we observed a significant decrease in overall injectable antimicrobials during 2013–2019 (Figures 3, 4; Appendix Figure 14). For feed, we observed a decrease in the use of macrolides, penicillins, streptogramins, and a significant increase in the use of bacitracins and orthosomycins (Figures 3, 4; Appendix Figure 16).

### Antimicrobial Use and AMR Analysis by Antimicrobial Class

**Figure 5:**
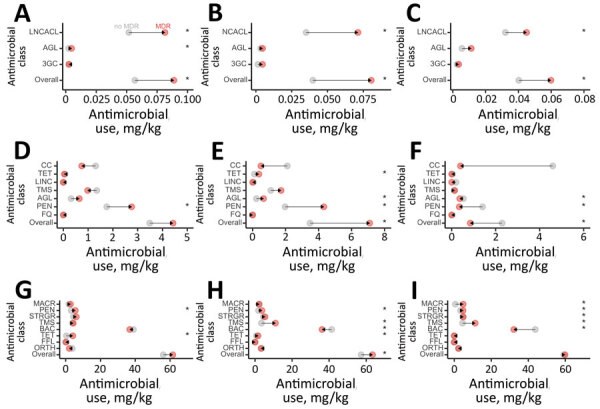
Mean antimicrobial use through injection, water, and feed in broiler chicken flocks where *Salmonella*, *Escherichia coli*, and *Campylobacter* were isolated, Canada, 2013–2019. A) *Salmonella*; B) *Escherichia coli*; C) *Campylobacter.* Route of administration in each panel: top, in ovo or subcutaneous injections; middle, water; bottom, feed. Arrows represent directionality from no multidrug resistance (MDR; gray) to MDR (red). Asterisks (*) indicates p<0.05, obtained from mixed effects logistic regression including antimicrobial use as fixed effect and flock and veterinarian identification as random effects. AGL, aminoglycoside; BAC, bacitracin; CC, chemical coccidiostats; FFL, flavophospholipid; FQ, fluoroquinolone; LINC, lincomycin; LNCACL, lincosamides; MACR, macrolide; ORTH, orthomycin; PEN, penicillin; STRGR, streptogramin; TET, tetracycline; TMS, trimethropim-sulfonamides; 3GC, third-generation cephalosporin.

Flocks from which multidrug-resistant (MDR) *Salmonella* was isolated (n = 79 of 604 total flocks) had significantly higher median overall AMU compared with flocks where no MDR *Salmonella* was identified. Specifically, MDR flocks had significantly higher use of injectable lincosamide-aminocyclitol combinations (Figure 5; Appendix Figure 5), penicillins through water (Figure 5; Appendix Figure 6), and penicillins and tetracyclines through feed (Figure 5; Appendix Figure 7). Flocks from which MDR *E. coli* was isolated (n = 444/928) also had significantly higher median overall AMU. Most important, these flocks had significantly higher use of lincosamide-aminocyclitol combinations in ovo or subcutaneously at the hatcheries (Figure 5; Appendix Figure 11); tetracyclines, aminoglycosides, and penicillins through water (Figure 5; Appendix Figure 12); and penicillins, trimethoprim/sulfonamide combinations, bacitracins, and tetracyclines through feed (Figure 5; Appendix Figure 13). Flocks from which MDR *Campylobacter* was isolated (n = 30/218) also had significantly higher median overall AMU. Specifically, these flocks had significantly higher use of injectable lincosamides (Figure 5; Appendix Figure 17); used significantly more aminoglycosides and penicillins through water (Figure 5; Appendix Figure 18); and used significantly more macrolides, penicillins, streptogramins, trimethoprim/sulfonamide combinations, and bacitracins through feed (Figure 5; Appendix Figure 19).

## Discussion

Our study examined AMU trends in broiler chicken production in Canada along with AMR trends in important foodborne bacteria. A reduction in both AMR and AMU was observed across most antimicrobials and classes during 2013–2019. The temporal reduction in AMU reflected the implementation of the Chicken Farmers of Canada’s AMU Reduction Initiative. This AMU stewardship program involved the elimination of the preventive use of certain antimicrobial classes in a stepwise manner (*13*). Results from this work indicate that a decrease in AMU contributed to a decrease in AMR over time for some antimicrobial drugs; however, increased AMR to streptomycin and tetracycline in *Salmonella* isolates, an increase in AMR to gentamicin and nalidixic acid in *E. coli* isolates, and an increase in AMR to nalidixic acid in *Campylobacter* were observed. We detected an increase in the use of aminoglycosides through water over time, which possibly contributed to the rise in *Salmonella* and *E. coli* aminoglycoside resistance. Historically, the administration of antimicrobials through water was largely for treatment of diseases such as those associated with avian pathogenic *E. coli* (*14*). Thus, this finding suggests that in addition to the elimination of hatchery-level use, reduced preventive AMU through feed potentially resulted in increased frequency of infectious diseases, thereby increasing the need for AMU through water for disease treatment. 

The overall rise the number of classes *Salmonella* isolates were resistant to in 2019 should also be put in perspective with the serotypes identified on farms. The mean proportion of *Salmonella* Kentucky relative to total *Salmonella* isolates increased in 2019 (Appendix Figure 20). Previous work has shown that *Salmonella* Kentucky frequently carries genes conferring resistance to tetracyclines and aminoglycosides (*23*). Therefore, the temporal trends in resistance to these antimicrobial classes could reflect the shift in *S. enterica* serotypes (*24*). Trends in *Salmonella* serotypes and AMR prevalence in poultry in Canada were studied recently (*25*), showing, similar to our results, that different *Salmonella* serotypes carry different resistance profiles that influence the overall prevalence of resistance. In Canada, passive surveillance in poultry frequently detects *Salmonella* Kentucky (*14*). This serotype is 1 of the etiologic agents of enteric disease and high rates of illness in broiler chickens in Egypt (*26*); however, in Canada, although this serovar was the second-most frequently isolated serovar from passive surveillance, its clinical importance has not yet been determined (*14*). Further studies should estimate whether reduced prophylactic AMU affects serotype diversity and assess whether the *Salmonella* Kentucky lineages circulating in poultry in Canada have clinical impact in broilers. In *Salmonella*-positive flocks, >1 serovar was isolated from a single flock. The serovar isolated from a single sample is generally supposed to represent the most predominant serovar. To reduce potential underestimation of serovar diversity within a flock, CIPARS/FoodNet Canada routinely cultures each sample (4 total).

The study shows that the injection of antimicrobials in ovo or subcutaneously at hatcheries is significantly associated with resistance in foodborne bacteria on the farm. The progressive elimination of AMU administered through injection (ceftiofur in 2014 then gentamicin and lincomycin/spectinomycin at the end of 2018) might have largely contributed to the observed decrease in AMR. In Canada, the injection in ovo or subcutaneously at the hatcheries with ceftiofur was aimed at the prevention of omphalitis caused by *E. coli.* Since 2005, and after the partial voluntary restriction of its use, a decline in the prevalence of third-generation cephalosporin-resistant *Salmonella* Heidelberg isolates in retail chicken was observed (*8*). Moreover, a reduction of AmpC-associated resistance genes was observed in *E. coli* after the elimination of preventive use in 2014, the second cessation of use nationally (*27*,*28*). We found a decrease not only of cephalosporin resistance (ceftriaxone and cefoxitin) but also ampicillin resistance in *Salmonella* and *E. coli* during 2013–2019. Therefore, decreased use of ceftiofur may have led to a concomitant decrease in resistance to ampicillin.

We did not identify resistance rate differences between antimicrobial-free and conventional farms. Some studies have shown that antimicrobial-free farms have significantly lower resistance rates for *Salmonella* (*29*) and *Campylobacter* (*30*) compared with conventional farms, whereas other studies do not report such differences (*7*,*31*). In our study, although AMR did not differ according to production system, we observed a significantly higher prevalence of *Salmonella* Heidelberg on conventional farms (Appendix Figure 1). Similarly, we observed a small to no effect of using the ideal method for cleaning and disinfection (*19*) on AMR. However, significantly higher prevalence of *Salmonella* Heidelberg and Kentucky (Appendix Figure 1) were found in flocks that did not use the ideal method of disinfection. This finding raises awareness of the larger impact of AMU even when hygiene methods are ideal, but more important, the shift in serotype composition might have affected AMR rate. For example, *Salmonella* Kentucky and Heidelberg have the highest frequencies of resistance to ciprofloxacin (*32*) and to cephalosporins (*33*). The differences in the number of antimicrobial-free (n = 286) and conventional (n = 1,612) farms included in this study may have affected the ability to detect significant differences in AMR levels between farm categories. As more producers transition to alternate production systems, drivers for AMR other than AMU could be further investigated.

In our study, an overall reduction in resistance levels in indicator and zoonotic foodborne bacteria of broiler chicken origin was successfully achieved in response to changes in AMU practices in broiler chickens in Canada during 2013–2019. Resistance to certain antimicrobial classes have emerged or increased; the increases may be associated with use of aminoglycosides through water for disease treatment, the shift in prevalence of different *Salmonella* serotypes over time, or both. Farms that use the ideal method of disinfection and farms classified as antimicrobial free had lower prevalence of *Salmonella* serotypes of higher public health importance, indicating that implementation of sanitation best practices and reduced AMU programs are beneficial. As evidenced by the AMR results, the removal of AMU exposures during the early stages of an animal’s life could further reduce AMR. Additional work should address the effect of reduction of AMU on production costs; relevant production indicators including bird morbidity, mortality, and feed-conversion rates; and bird welfare in broiler chicken farms in Canada. The emerging practices on the use of alternatives to antimicrobials (e.g., vaccines against *E. coli*, *Salmonella*, and gut health enhancers) also warrant further investigation. This additional information will provide future guidance for the progressive transition from the current AMU-dependent production systems to alternative and sustainable measures to promote animal health and productivity.

Appendix Additional information about reduction in antimicrobial use and resistance to *Salmonella*, *Campylobacter*, and *Escherichia coli* in broiler chickens, Canada, 2013–2019.
