# Supplementary material for: Reduction in Antimicrobial Use and Resistance to Salmonella, Campylobacter, and Escherichia coli in Broiler Chickens, Canada, 2013–2019
Source: Emerg Infect Dis. 2021 Sep;27(9):2434–44. doi: 10.3201/eid2709.204395 (PMC8386787; doi:10.3201/eid2709.204395)
Supplement: Appendix — Additional information about reduction in antimicrobial use and resistance to Salmonella, Campylobacter, and Escherichia coli in broiler chickens, Canada, 2013–2019. [file 20-4395-Techapp-s1.pdf]

Article DOI: https://doi.org/10.3201/eid2709.204395

# Reduction in Antimicrobial Use and Resistance to *Salmonella*, *Campylobacter*, and *Escherichia coli* in Broiler Chickens, Canada, 2013–2019

## Appendix

**Appendix Table 1.** Susceptibility testing breakpoints for *Salmonella*, *Campylobacter*, and *E. coli,* and mean (95% CI) percent resistance against each of the main antimicrobial classes tested for the three pathogens between 2013 and 2019*

| | Breakpoints (µg/mL) | | | |
|---|---|---|---|---|
| Antimicrobial | Susceptible | Intermediate | Resistant | % Resistance (95% CI) |
| *Campylobacter* (n = 769) | | | | |
| Azithromycin (AZM) | ≤2 | 4 | ≥8 | 3.77 (2.42, 5.12) |
| Erythromycin (ERY) | ≤8 | 16 | ≥32 | 3.77 (2.42, 5.12) |
| Florfenicol (FLR) | ≤4 | NA | NA | 0.00 (0.00, 0.00) |
| Tetracycline (TET) | ≤4 | 8 | ≥16 | 38.75 (35.31, 42.19) |
| Ciprofloxacin (CIP) | ≤1 | 2 | ≥4 | 16.51 (13.89, 19.14) |
| Gentamicin (GEN) | ≤2 | 4 | ≥8 | 0.00 (0.00, 0.00) |
| Clindamycin (CLI) | ≤2 | 4 | ≥8 | 2.08 (1.07, 3.10) |
| Nalidixic Acid (NAL) | ≤16 | 32 | ≥64 | 16.38 (13.77, 19.00) |
| *E. coli* (n = 3,671) | | | | |
| Amoxicillin-clavulanic acid (AMC) | ≤8/4 | 16/8 | ≥32/16 | 14.11 (12.98, 15.23) |
| Chloramphenicol (CHL) | ≤8 | 16 | ≥32 | 5.86 (5.09, 6.62) |
| Gentamicin | ≤4 | 8 | ≥16 | 18.41 (17.16, 19.67) |
| Sulfisoxazole (SSS) | ≤256 | NA | ≥512 | 39.42 (37.83, 41.00) |
| Ampicillin (AMP) | ≤8 | 16 | ≥32 | 40.53 (38.95, 42.12) |
| Ciprofloxacin | ≤0.06 | 0.12–0.5 | ≥1 | 0.29 (0.12, 0.47) |
| Streptomycin (STR) | ≤16 | NA | ≥32 | 46.33 (44.72, 47.94) |
| Trimethoprim-sulfonamides (SXT) | ≤2/38 | NA | ≥4/76 | 16.10 (14.91, 17.29) |
| Azithromycin | ≤16 | NA | ≥32 | 0.41 (0.20, 0.61) |
| Ceftriaxone (CRO) | ≤1 | 2 | ≥4 | 13.73 (12.62, 14.84) |
| Cefoxitin (FOX) | ≤8 | 16 | ≥32 | 14.06 (12.93, 15.18) |
| Nalidixic Acid | ≤16 | NA | ≥32 | 6.18 (5.40, 6.96) |
| Tetracycline | ≤4 | 8 | ≥16 | 46.82 (45.21, 48.44) |
| *Salmonella* (n = 1,898) | | | | |
| Amoxicillin-clavulanic acid | ≤8/4 | 16/8 | ≥32/16 | 11.43 (10.00, 12.86) |
| Chloramphenicol | ≤8 | 16 | ≥32 | 0.42 (0.13, 0.71) |
| Gentamicin | ≤4 | 8 | ≥16 | 1.32 (0.80, 1.83) |
| Sulfisoxazole | ≤256 | NA | ≥512 | 6.48 (5.37, 7.59) |
| Ampicillin | ≤8 | 16 | ≥32 | 11.43 (10.00, 12.86) |
| Ciprofloxacin | ≤0.06 | 0.12–0.5 | ≥1 | 0.16 (0.00, 0.33) |
| Streptomycin | ≤16 | NA | ≥32 | 43.57 (41.34, 45.80) |
| Azithromycin | ≤16 | NA | ≥32 | 0.00 (0.00, 0.00) |
| Ceftriaxone | ≤1 | 2 | ≥4 | 11.49 (10.05, 12.92) |
| Cefoxitin | ≤8 | 16 | ≥32 | 10.22 (8.86, 11.58) |
| Nalidixic Acid | ≤16 | NA | ≥32 | 2.32 (1.64, 3.00) |
| Tetracycline | ≤4 | 8 | ≥16 | 44.73 (42.50, 46.97) |

*Breakpoints stablished by Clinical and Laboratory Standards Institute were used when available. If unavailable, Not Applicable (NA) was assigned.

**Appendix Table 2.** Variables evaluated by the LASSO regression.

| Variable | Distribution | Levels | Unit |
|---|---|---|---|
| Production system | Binary | Conventional, antimicrobial-free* | NA |
| Disinfection method | Binary | Ideal,† Other | NA |
| All-in-all-out | Binary | Yes, No | NA |
| Province | Categorical (5 levels) | Alberta, Saskatchewan, Ontario, British Columbia, and Québec | NA |
| Year of sample collection | Categorical (7 levels) | 2013, 2014, 2015, 2016, 2017, 2018, 2019 | NA |
| AMU *in ovo* | Continuous | NA | mg/kg |
| AMU *in ovo* | Binary | Yes, No | NA |
| AMU via feed | Continuous | NA | mg/kg |
| AMU via feed | Binary | Yes, No | NA |
| AMU via water | Continuous | NA | mg/kg |
| AMU via water | Binary | Yes, No | NA |

*Farms that are not exposed to medically important antimicrobials, but that may allow the use of ionophore and chemical coccidiostats.
†Ideal method for cleaning and disinfection recommended by the World Organization for Animal Health.

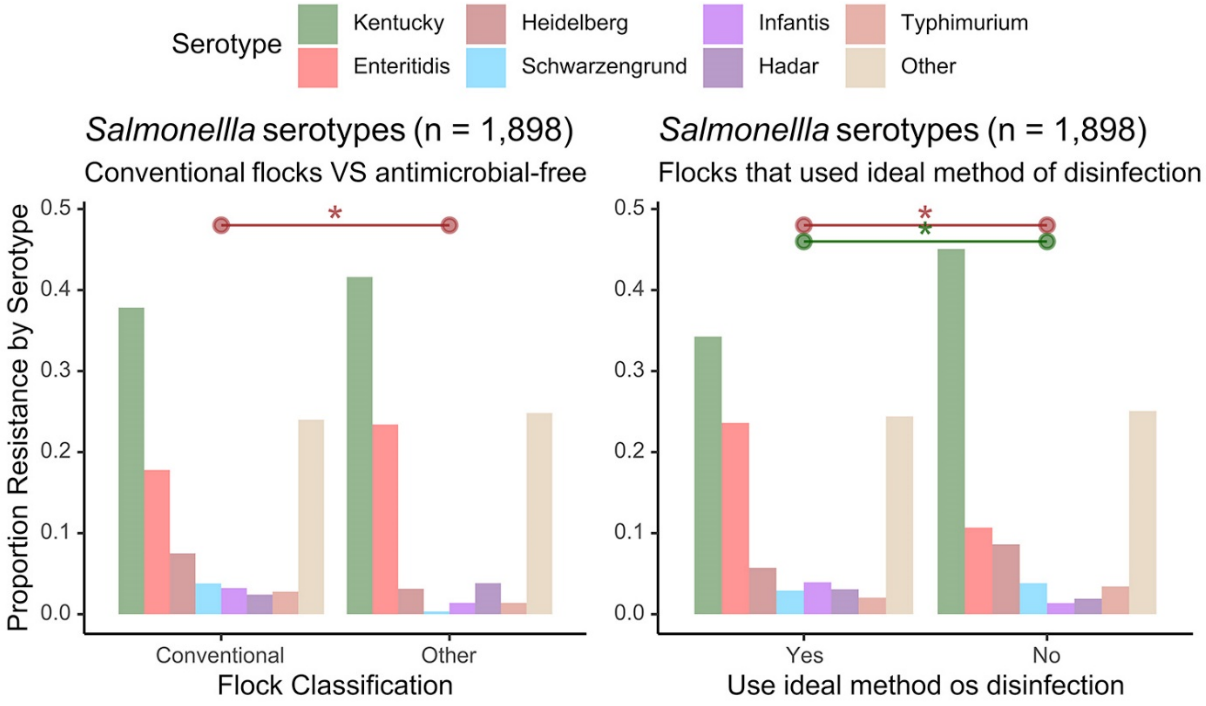


**Appendix Figure 1.** Differences in *Salmonella* (n = 1,898) serotypes proportions in chicken flocks that are classified as conventional versus antimicrobial-free; and between farms reporting to have used the ideal method of disinfection before sample collection versus farms that did not use this method of disinfection. Color-coded * by serotype represents p<0.05 using Z test to compare two population proportions.

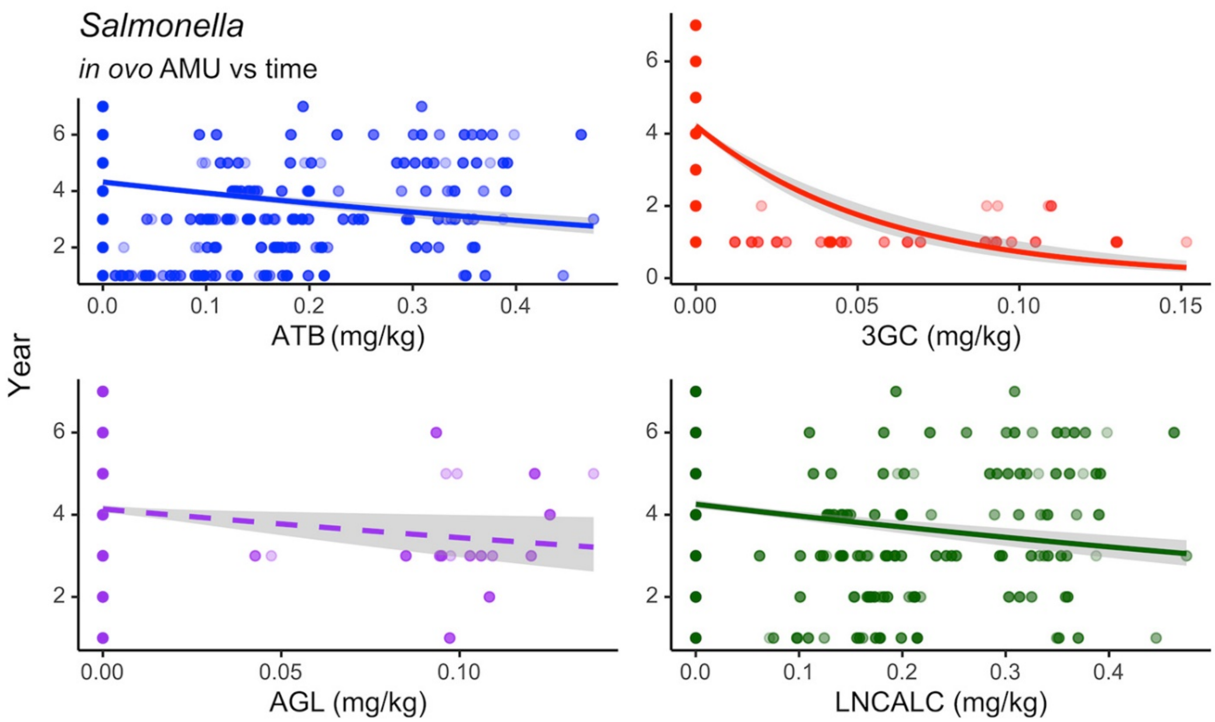


**Appendix Figure 2.** Generalized linear models demonstrating the change in overall (log-transformed mg/kg) use of antimicrobials in flocks where *Salmonella* was isolated, and stratified by the use of third-generation cephalosporins, aminoglycosides and lincosamides *in ovo* over time. Continuous lines represent significance (p<0.05).

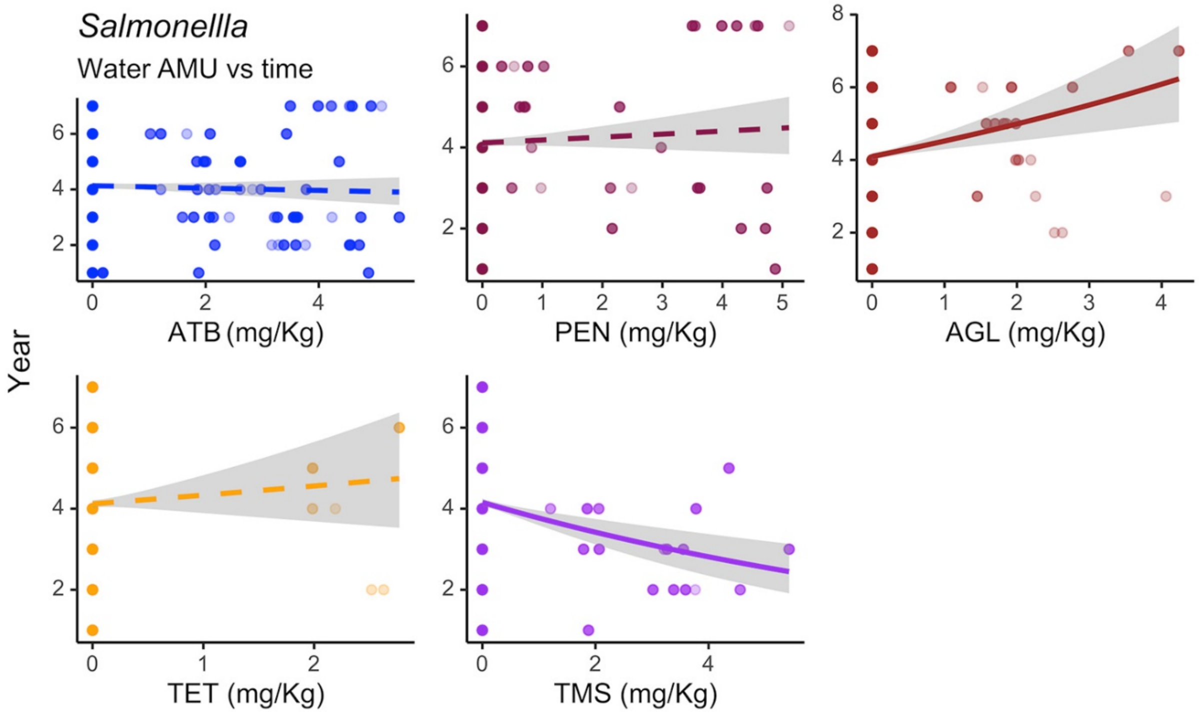


**Appendix Figure 3.** Generalized linear models demonstrating the change in overall (log-transformed mg/kg) use of antimicrobials in flocks where *Salmonella* was isolated, and stratified by the use of penicillins, aminoglycosides, tetracyclines, and trimethoprim-sulfonamides via water over time. Continuous lines represent significance ($p<0.05$).

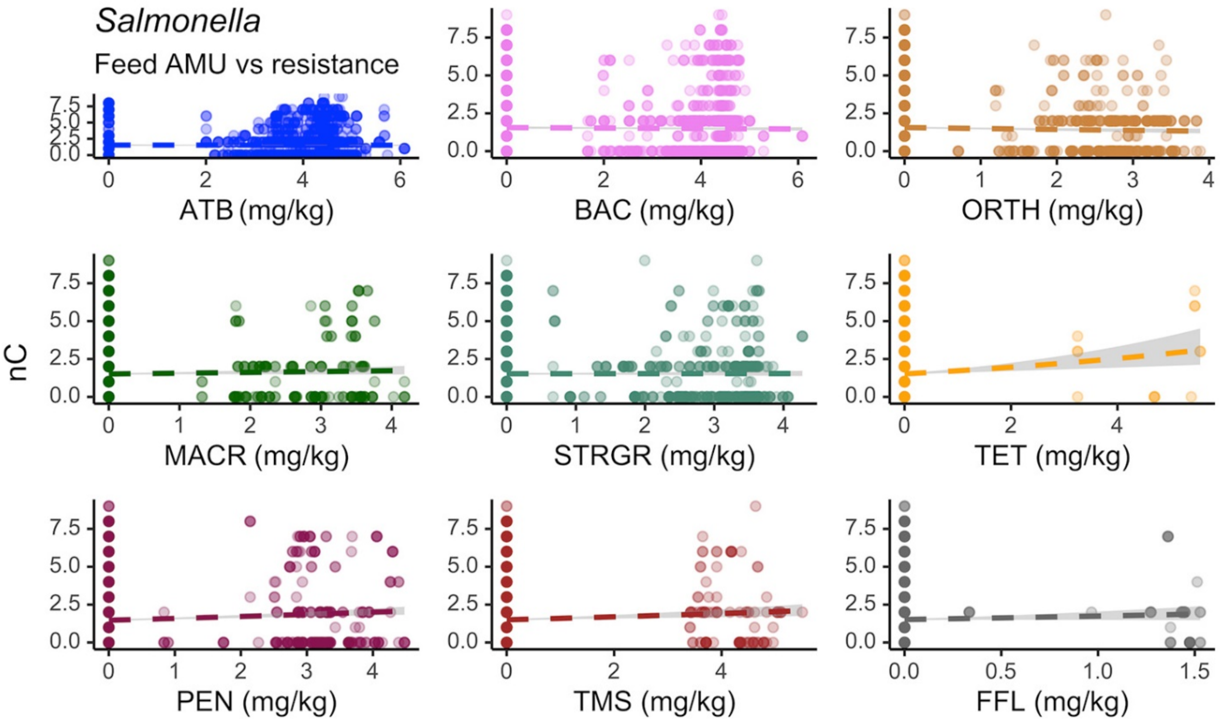


**Appendix Figure 4.** Generalized linear models demonstrating the change in overall (log-transformed mg/kg) use of antimicrobials in flocks where *Salmonella* was isolated, and stratified by the use of bacitracins, orthosomycins, macrolides, streptogramins, tetracyclines, penicillins, trimethoprim-sulfonamides and flavophospholipids via feed over time. Continuous lines represent significance ($p<0.05$).

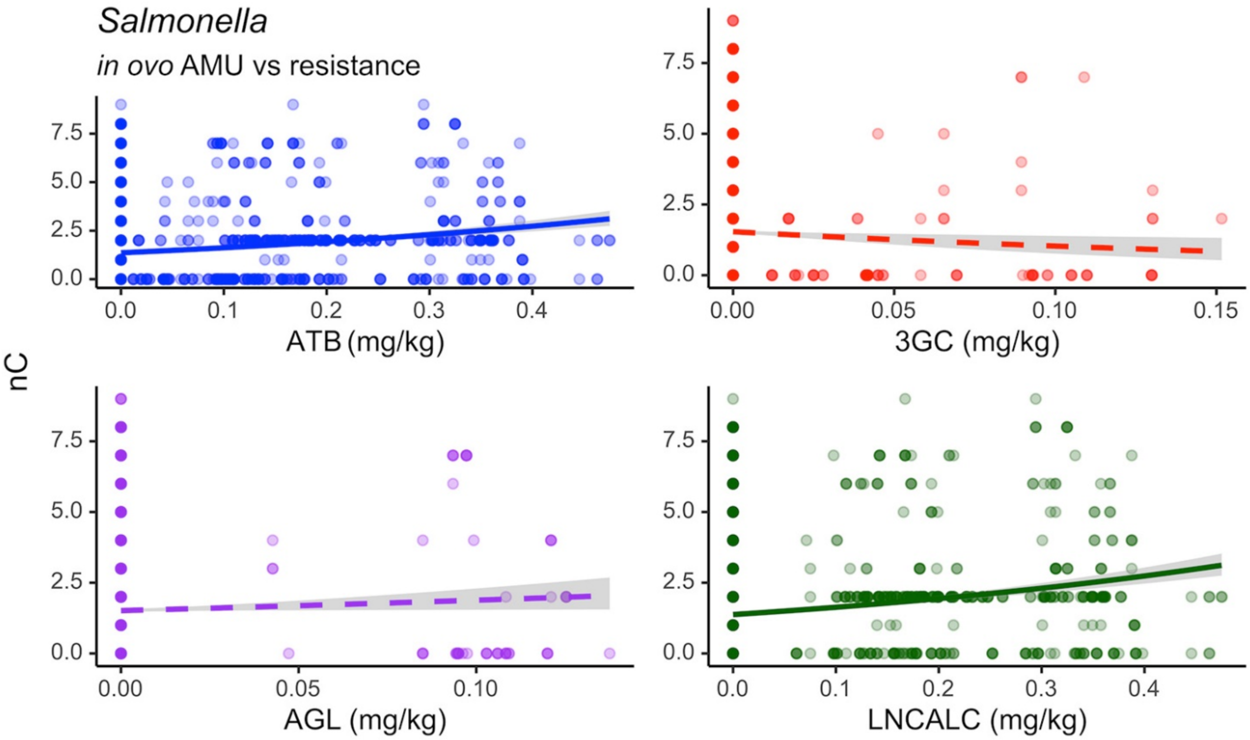


**Appendix Figure 5.** Generalized linear models illustrating the change in the number of antimicrobial classes a *Salmonella* isolate is resistant to (nC) in response to overall (log transformed mg/kg) use of antimicrobials, and stratified by the use of third generation cephalosporins, aminoglycosides, and lincosamides *in ovo*. Continuous lines represent significance ($p<0.05$).

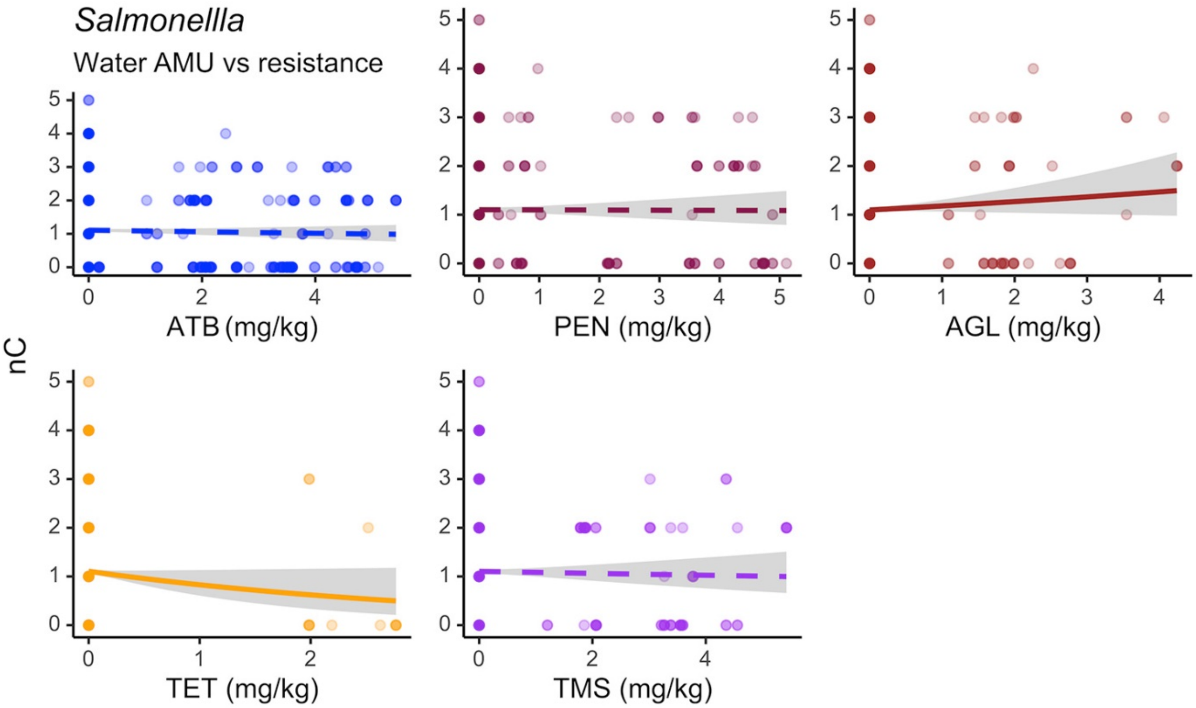


**Appendix Figure 6.** Generalized linear models illustrating the change in the number of antimicrobial classes a *Salmonella* isolate is resistant to (nC) in response to overall use of antimicrobials (log transformed mg/kg), and stratified by the use of penicillins, aminoglycosides, tetracyclines, and trimethoprim-sulfonamide combinations via water. Continuous lines represent significance ($p<0.05$).

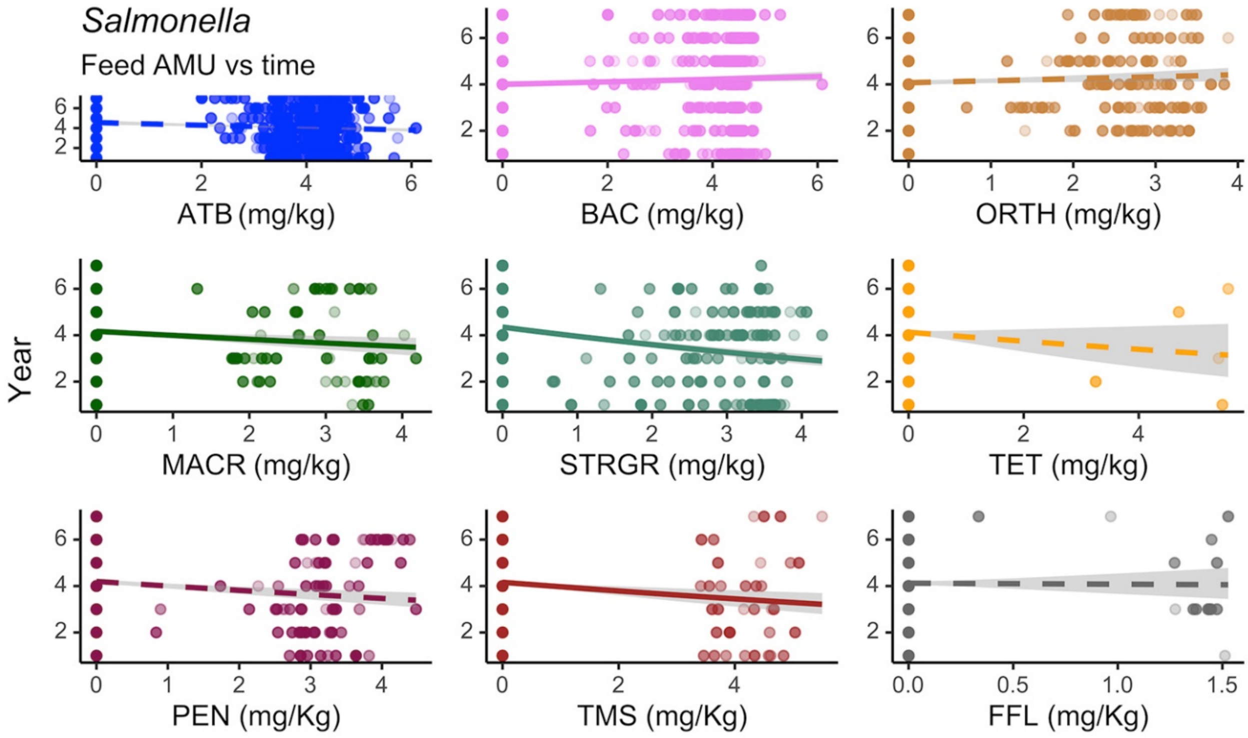


**Appendix Figure 7.** Generalized linear models illustrating the change in the number of antimicrobial classes a *Salmonella* isolate is resistant to (nC) in response to overall use of antimicrobials (log transformed mg/kg), and stratified by the use of bacitracins, orthosomycins, macrolides, streptogramins, tetracyclines, penicillins, trimethoprim-sulfonamides and flavophospholipids via feed. Continuous lines represent significance ($p<0.05$).

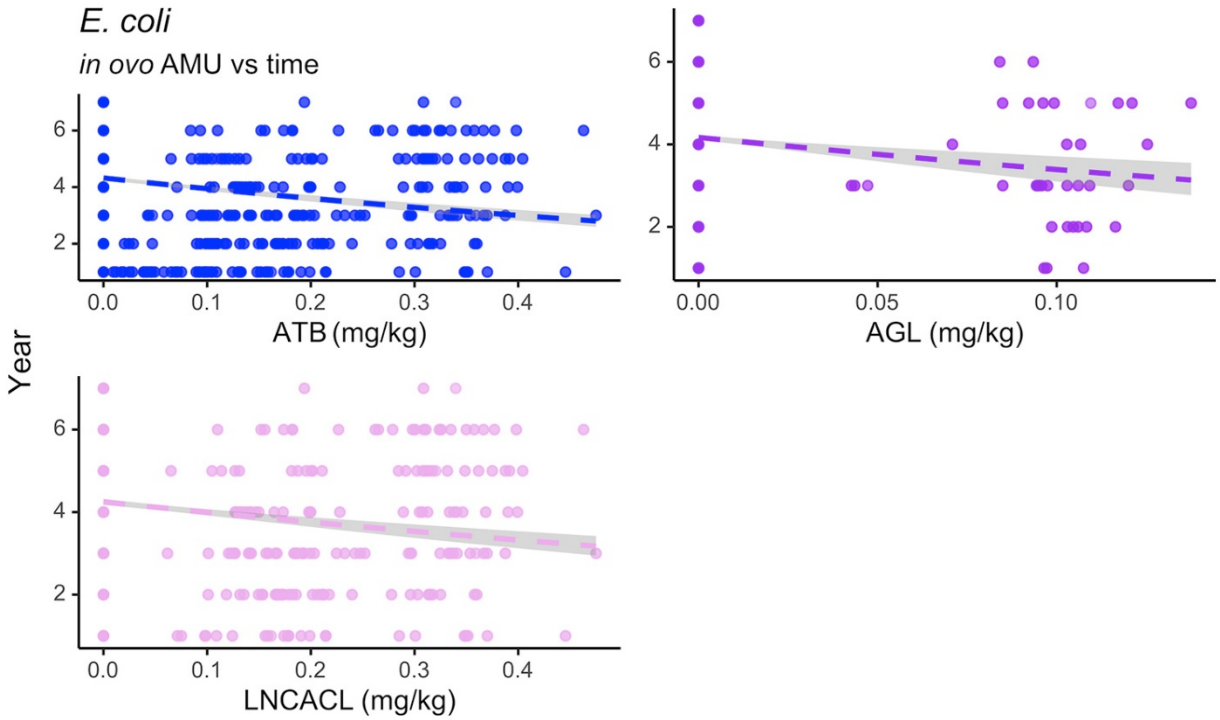


**Appendix Figure 8.** Generalized linear models demonstrating the change in overall (log-transformed mg/kg) use of antimicrobials in flocks where *E. coli* was isolated, and stratified by the use of aminoglycosides and lincosamides *in ovo* over time. Continuous lines represent significance ($p<0.05$).

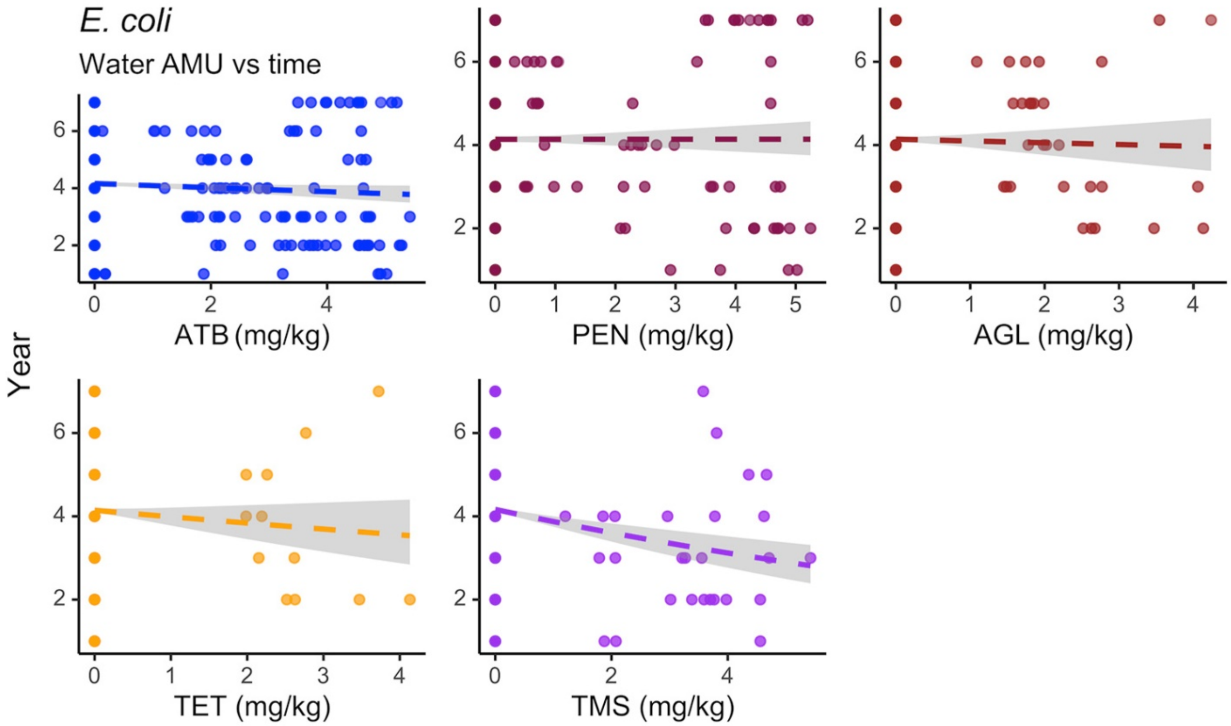


**Appendix Figure 9.** Generalized linear models demonstrating the change in overall (log-transformed mg/kg) use of antimicrobials in flocks where *E. coli* was isolated, and stratified by the use of penicillins, aminoglycosides, tetracyclines, and trimethoprim-sulfonamide combinations via water over time. Continuous lines represent significance ($p<0.05$).

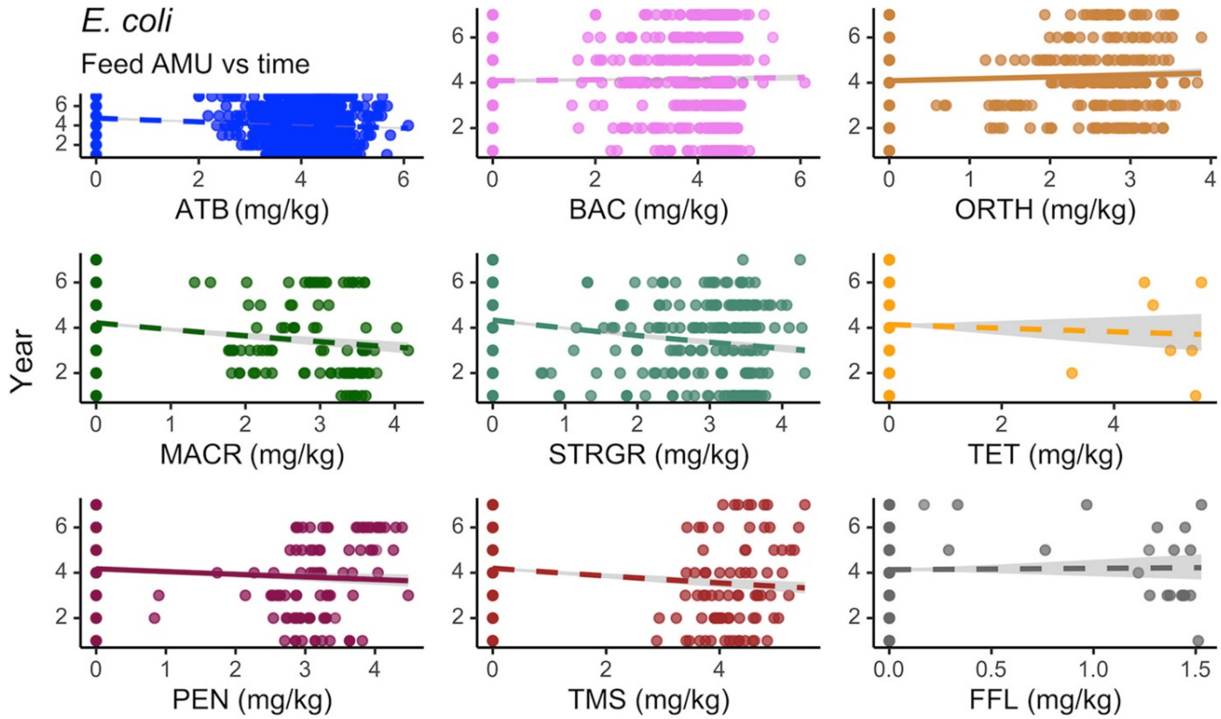


**Appendix Figure 10.** Generalized linear models demonstrating the change in overall (log-transformed mg/kg) use of antimicrobials in flocks where *E. coli* was isolated, and stratified by the use of bacitracins, orthosomycins, macrolides, streptogramins, tetracyclines, penicillins, trimethoprim-sulfonamide combinations, and flavophospholipids via feed over time. Continuous lines represent significance ($p<0.05$).

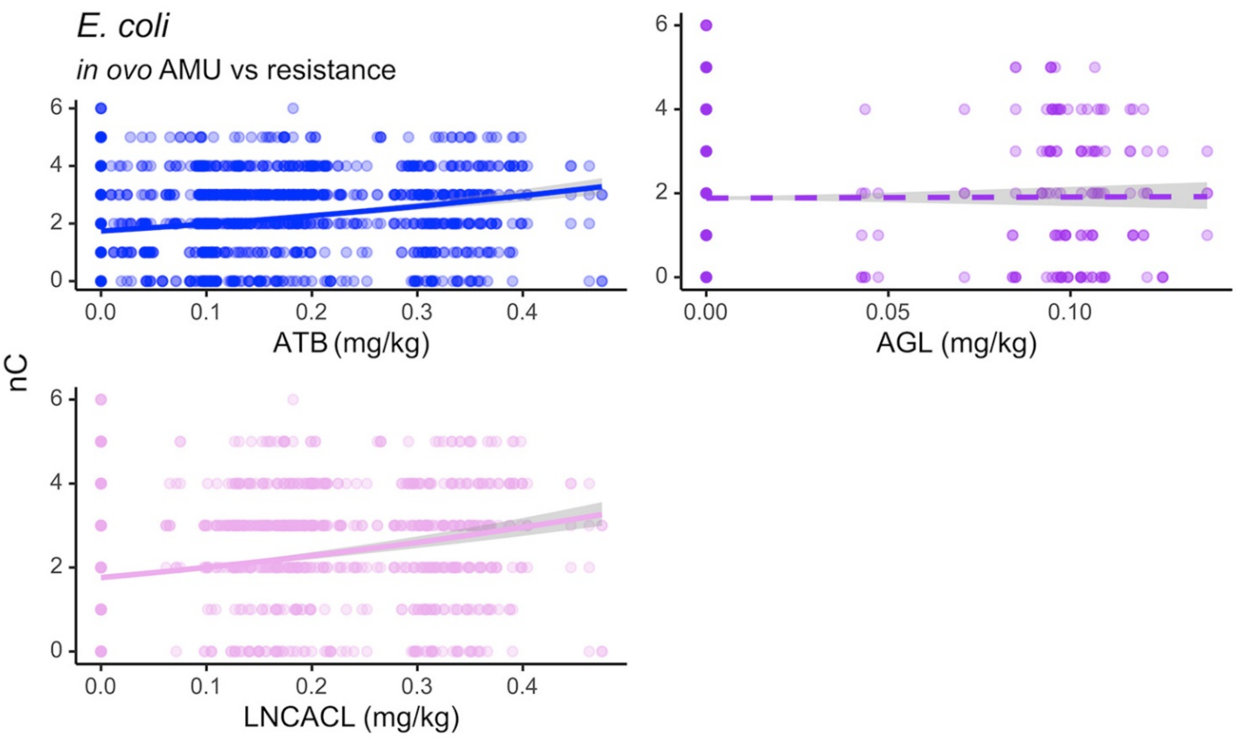


**Appendix Figure 11.** Generalized linear models illustrating the change in the number of antimicrobial classes a *E. coli* isolate is resistant to (nC) in response to overall use of antimicrobials (log transformed mg/kg), and stratified by the use of aminoglycosides and lincosamides *in ovo*. Continuous lines represent significance ($p<0.05$).

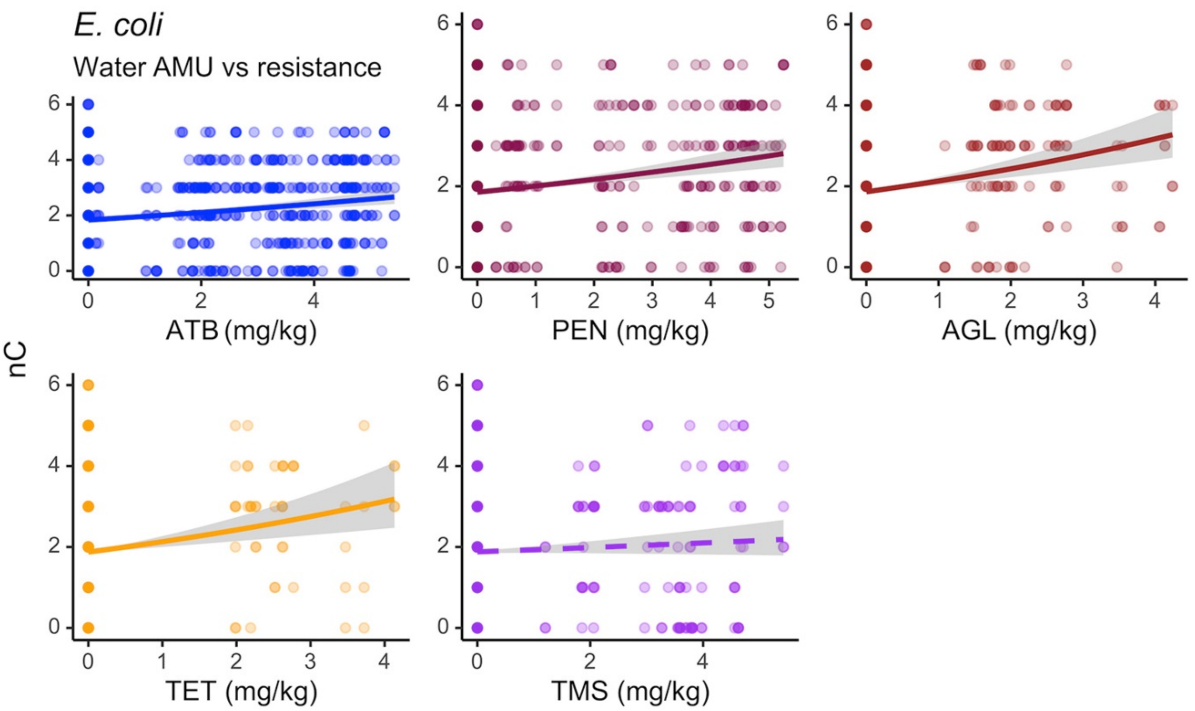


**Appendix Figure 12.** Generalized linear models illustrating the change in the number of antimicrobial classes a *E. coli* isolate is resistant to (nC) in response to overall use of antimicrobials (log transformed mg/kg), and stratified by the use of penicillins, aminoglycosides, tetracyclines, and trimethoprim-sulfonamide combinations via water. Continuous lines represent significance ($p<0.05$).

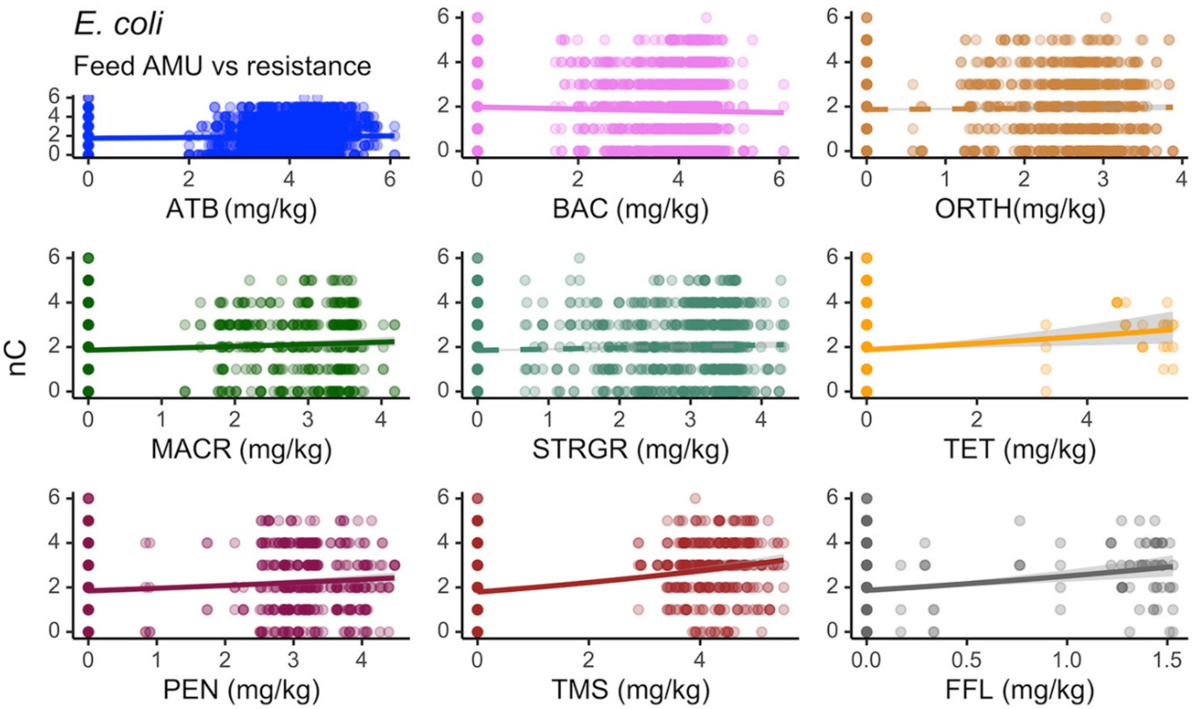


**Appendix Figure 13.** Generalized linear models illustrating the change in the number of antimicrobial classes a *E. coli* isolate is resistant to (nC) in response to overall use of antimicrobials (log transformed mg/kg), and stratified by the use of bacitracins, orthosomycins, macrolides, streptogramins, tetracyclines, penicillins, trimethoprim-sulfonamides, and flavophospholipids via feed. Continuous lines represent significance ($p<0.05$).

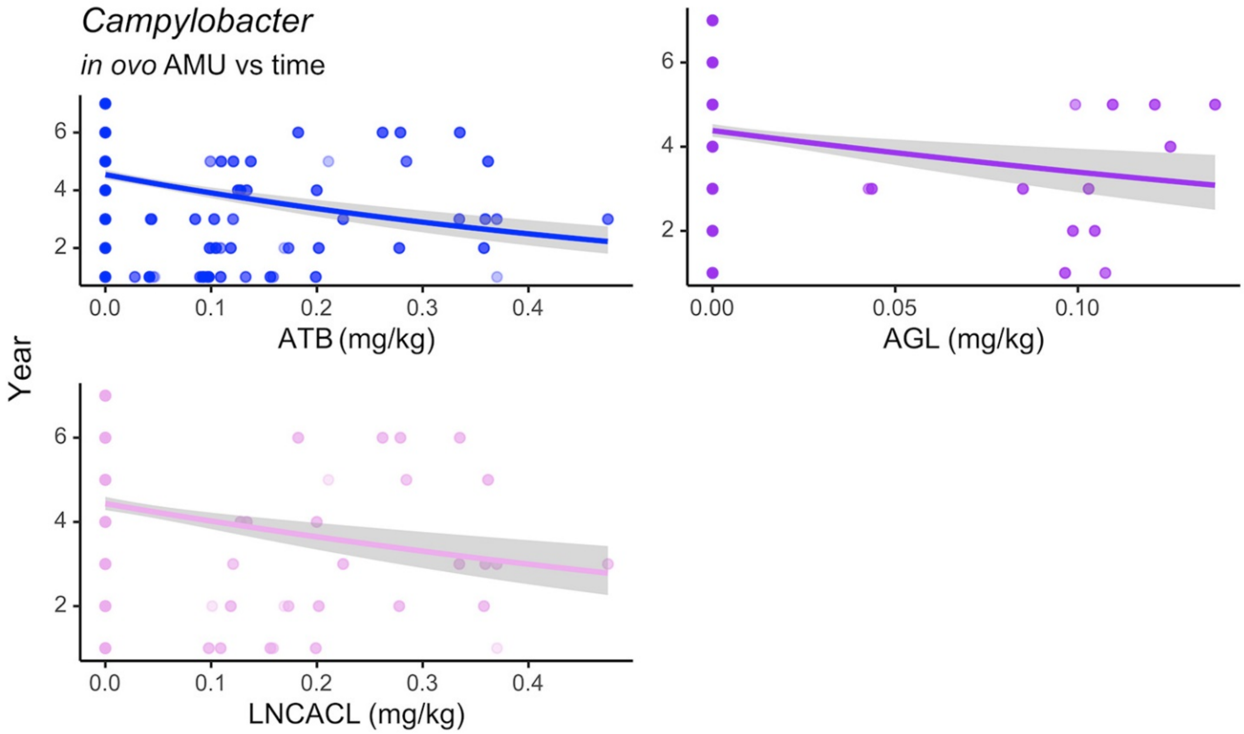


**Appendix Figure 14.** Generalized linear models demonstrating the change in overall (log-transformed mg/kg) use of antimicrobials in flocks where *Campylobacter* was isolated, and stratified by the use of aminoglycosides and lincosamides *in ovo* over time. Continuous lines represent significance ($p<0.05$).

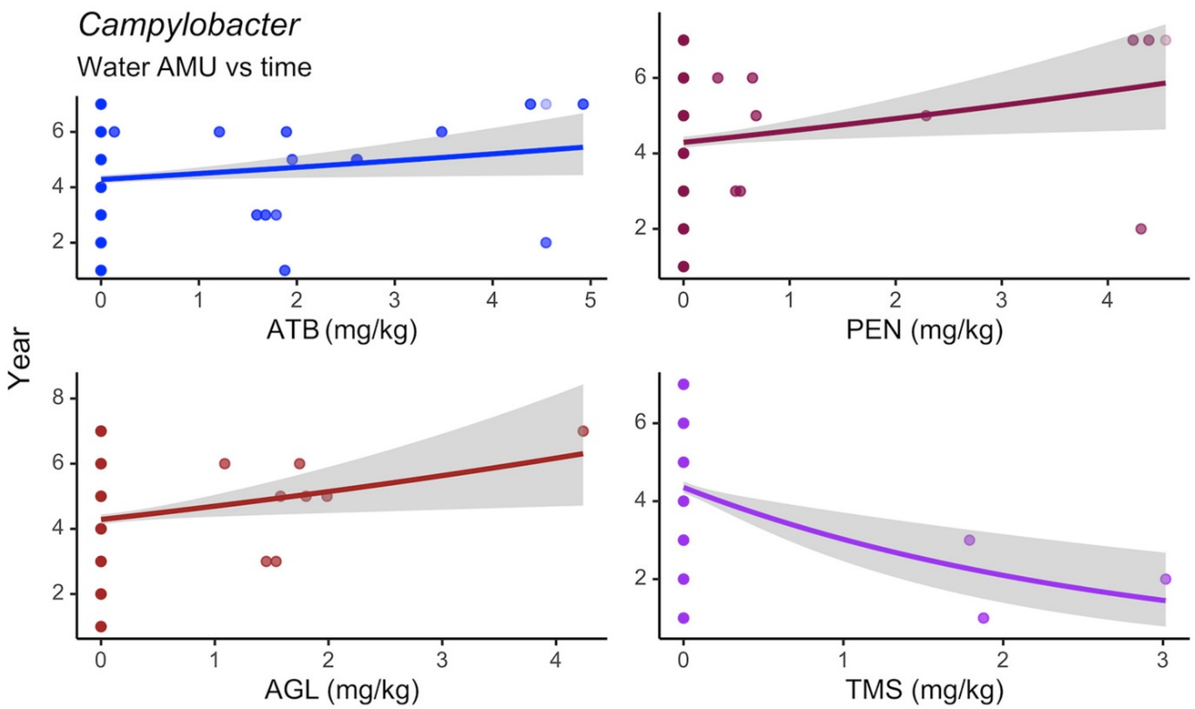


**Appendix Figure 15.** Generalized linear models demonstrating the change in overall (log-transformed mg/kg) use of antimicrobials in flocks where *Campylobacter* was isolated, and stratified by the use of penicillins, aminoglycosides, tetracyclines, and trimethoprim-sulfonamides via water over time. Continuous lines represent significance ($p<0.05$).

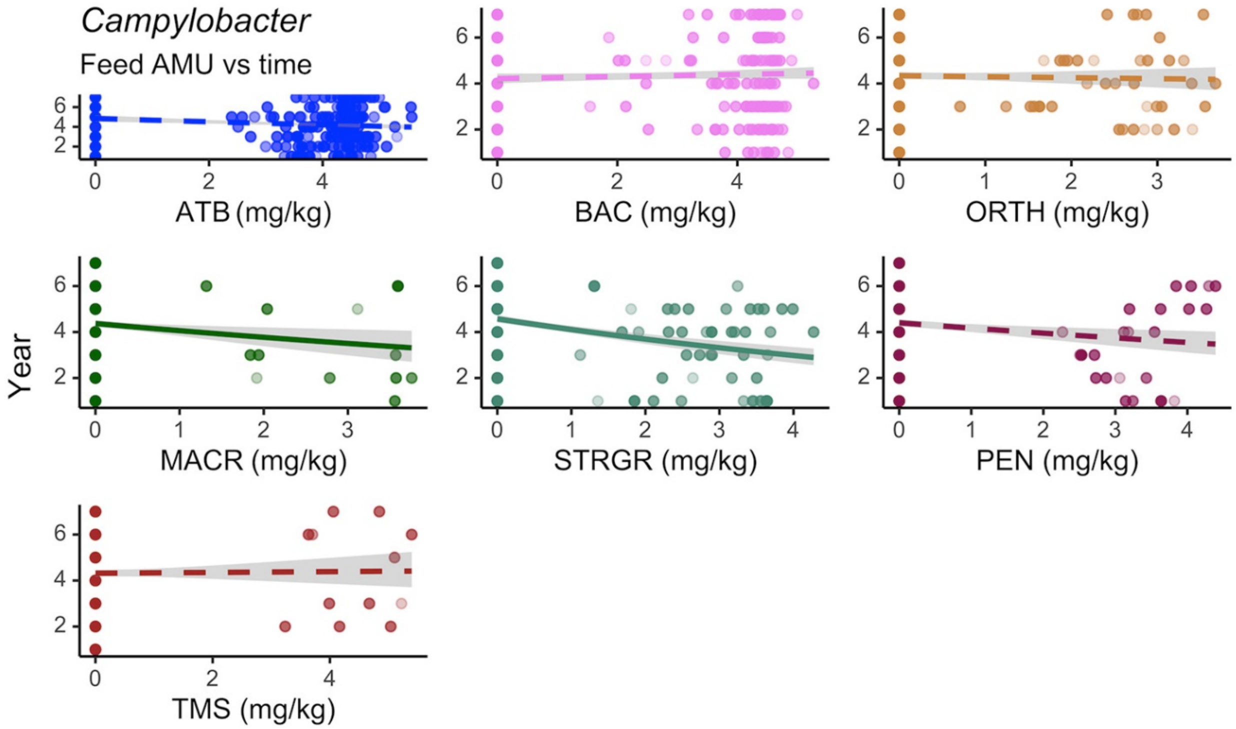


**Appendix Figure 16.** Generalized linear models demonstrating the change in overall (log-transformed mg/Kg) use of antimicrobials in flocks where *Campylobacter* was isolated, and stratified by the use of bacitracins, orthosomycins, macrolides, streptogramins, tetracyclines, penicillins, and trimethoprim-sulfonamide combinations via feed over time. Continuous lines represent significance ($p<0.05$).

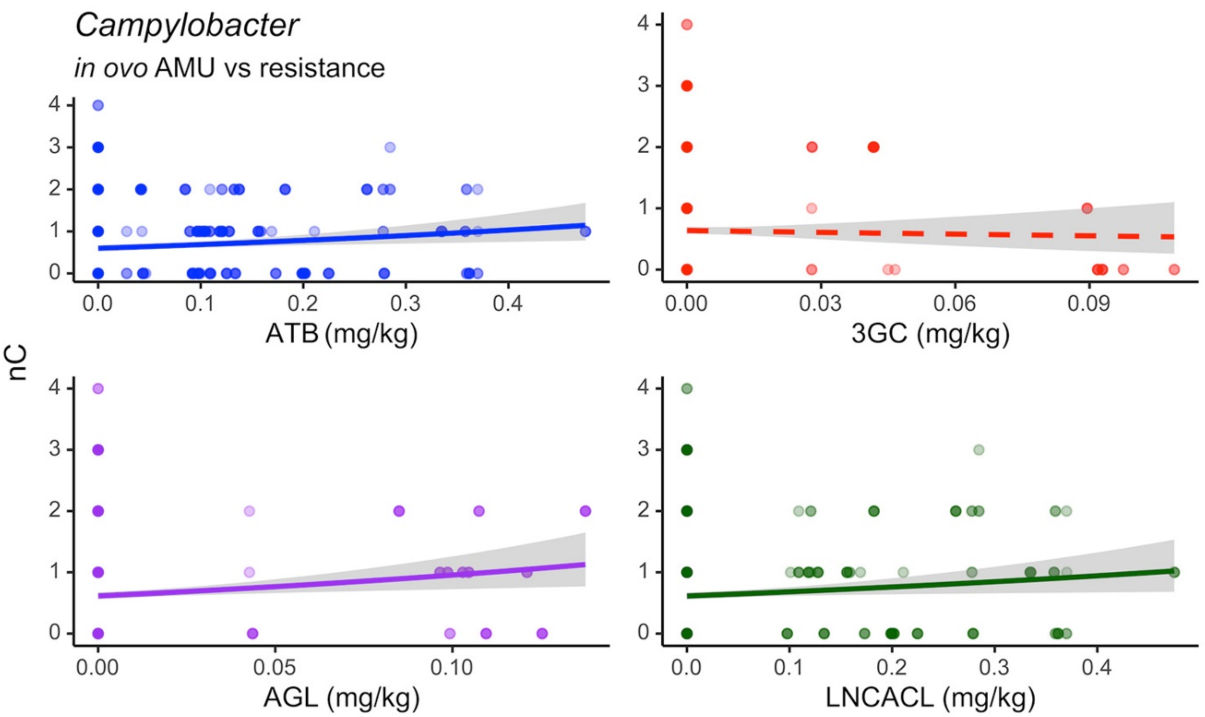


**Appendix Figure 17.** Generalized linear models illustrating the change in the number of antimicrobial classes a *Campylobacter* isolate is resistant to (nC) in response to overall use of antimicrobials (log transformed mg/kg), and stratified by the use of third generation cephalosporins, aminoglycosides, and lincosamides *in ovo*. Continuous lines represent significance (p<0.05).

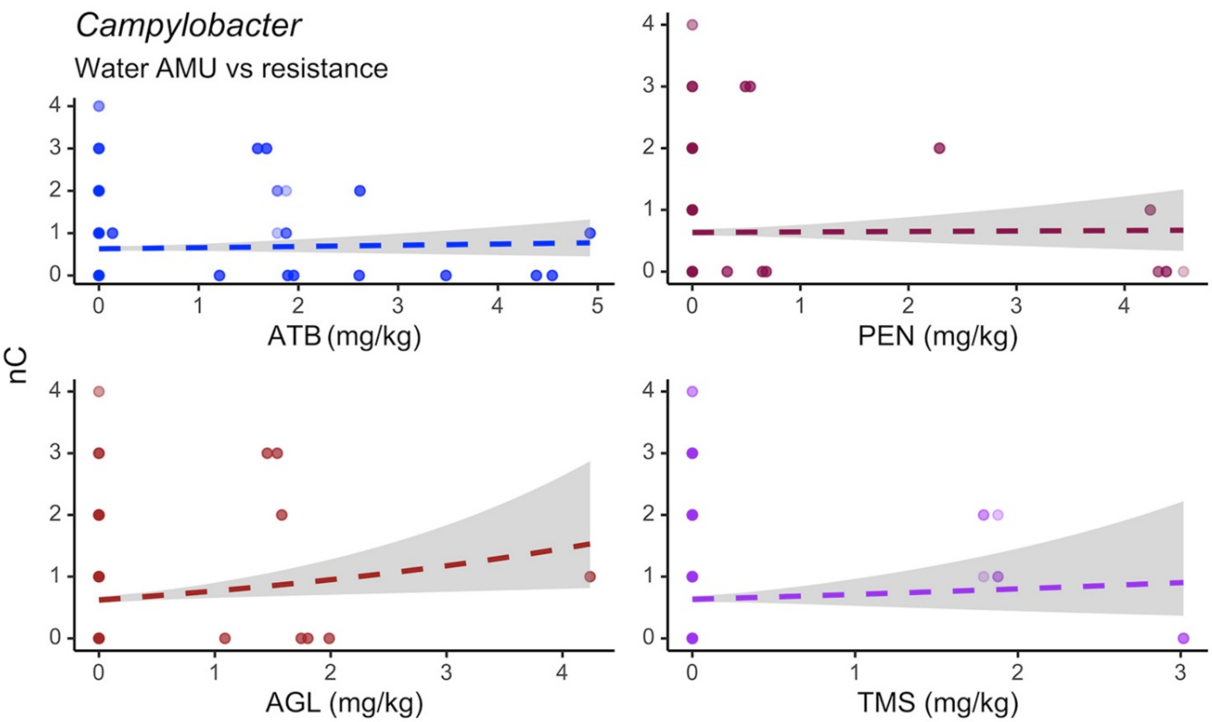


**Appendix Figure 18.** Generalized linear models illustrating the change in the number of antimicrobial classes a *Campylobacter* isolate is resistant to (nC) in response to overall use of antimicrobials (log transformed mg/kg), and stratified by the use of penicillins, and trimethoprim-sulfonamide combinations via water. Continuous lines represent significance ($p<0.05$).

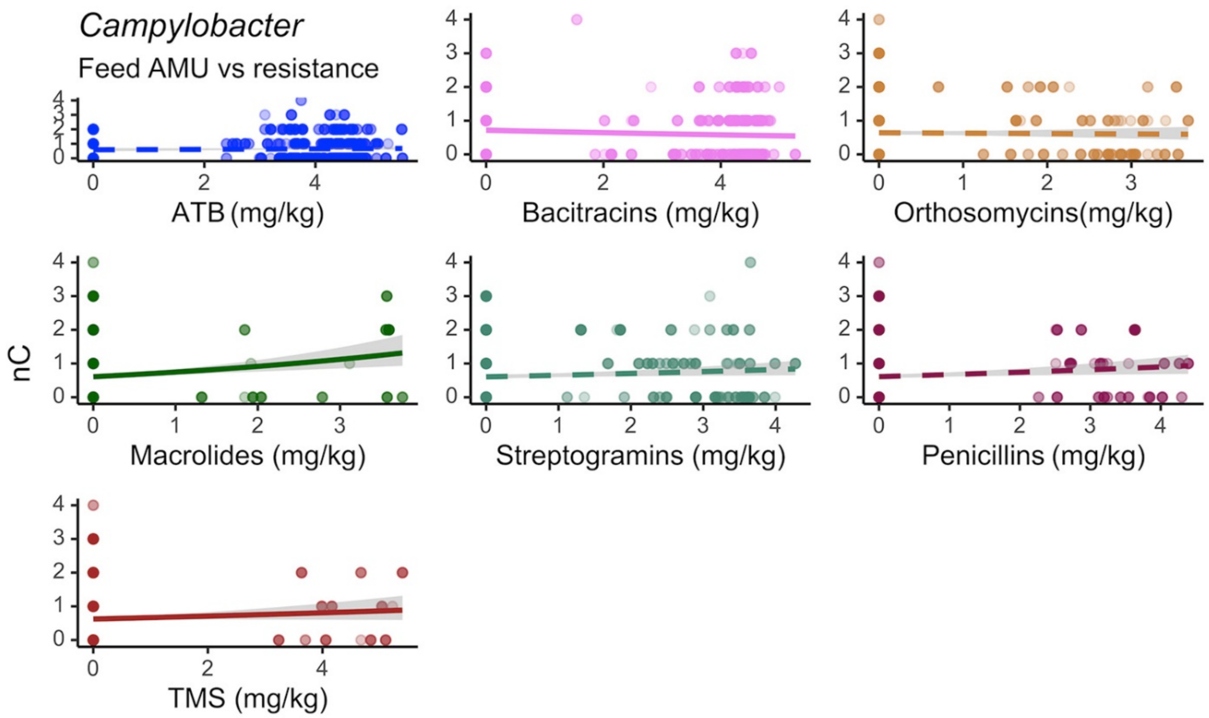


**Appendix Figure 19.** Generalized linear models illustrating the change in the number of antimicrobial classes a *Campylobacter* isolate is resistant to (nC) in response to overall use of antimicrobials (log transformed mg/kg), and stratified by the use of bacitracins, orthosomycins, macrolides, streptogramins, penicillins, and trimethoprim-sulfonamides via feed. Continuous lines represent significance ($p<0.05$).

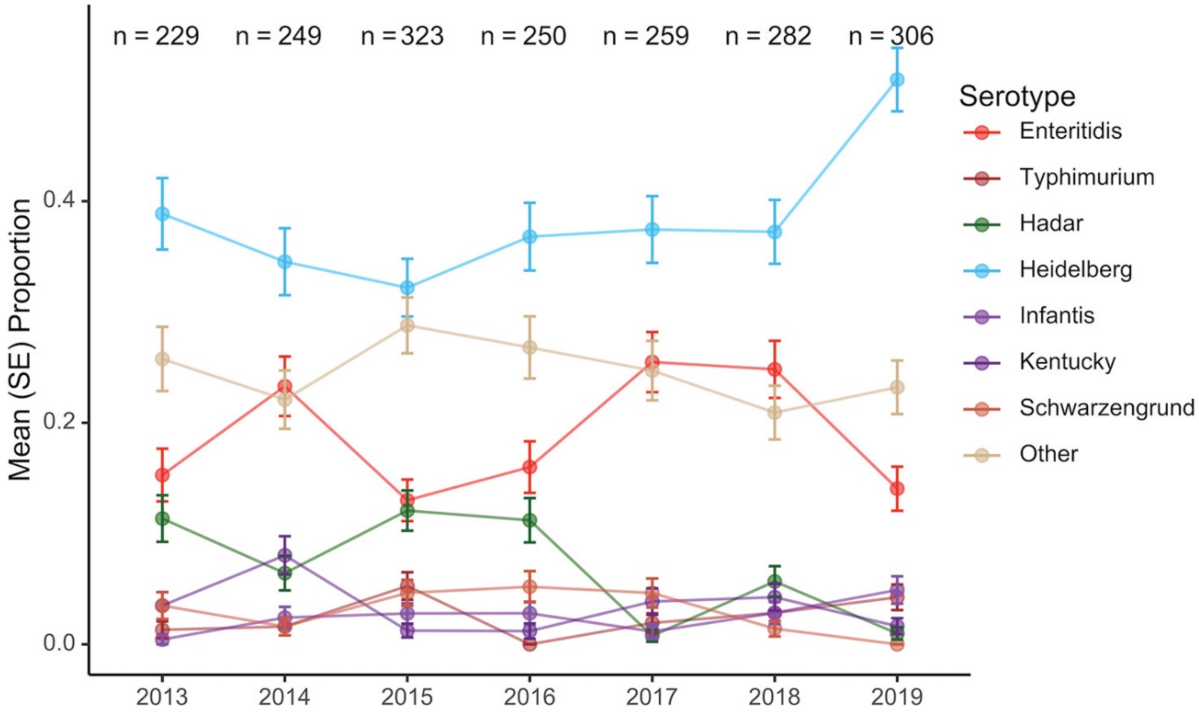


**Appendix Figure 20.** Mean proportion and standard errors of the *Salmonella* (n = 1,898) serotypes isolated from chicken flocks in Canada over the study period (2013–2019).
